# Thiophene α-Chain-End-Functionalized Oligo(2-methyl-2-oxazoline) as Precursor Amphiphilic Macromonomer for Grafted Conjugated Oligomers/Polymers and as a Multifunctional Material with Relevant Properties for Biomedical Applications

**DOI:** 10.3390/ijms23147495

**Published:** 2022-07-06

**Authors:** Anca-Dana Bendrea, Luminita Cianga, Gabriela-Liliana Ailiesei, Demet Göen Colak, Irina Popescu, Ioan Cianga

**Affiliations:** 1 Centre of Advanced Research in Bionanoconjugates and Biopolymers, “PetruPoni” Institute of Macromolecular Chemistry, 41 A, Grigore-GhicaVoda Alley, 700487 Iasi, Romania; anca.bendrea@icmpp.ro; 2 NMR Spectroscopy Department, “PetruPoni” Institute of Macromolecular Chemistry, 41 A, Grigore-GhicaVoda Alley, 700487 Iasi, Romania; gdarvaru@icmpp.ro; 3 Department of Chemistry, Faculty of Science and Letters, Istanbul Technical University, Maslak, 34469 Istanbul, Turkey; goende@itu.edu.tr; 4 Department of Natural Polymers, Bioactive and Biocompatible Materials, “PetruPoni” Institute of Macromolecular Chemistry, 41 A, Grigore-GhicaVoda Alley, 700487 Iasi, Romania; ipopescu@icmpp.ro

**Keywords:** electroactive macromonomers, oligo(2-methyl-2-oxazoline), amphiphiles, clusteroluminescence, polythiophenes

## Abstract

Because the combination of π-conjugated polymers with biocompatible synthetic counterparts leads to the development of bio-relevant functional materials, this paper reports a new oligo(2-methyl-2-oxazoline) (OMeOx)-containing thiophene macromonomer, denoted **Th-OMeOx**. It can be used as a reactive precursor for synthesis of a polymerizable 2,2’-3-OMeOx-substituted bithiophene by Suzuki coupling. Also a grafted polythiophene amphiphile with OMeOx side chains was synthesized by its self-acid-assisted polymerization (SAAP) in bulk. The results showed that **Th-OMeOx** is not only a reactive intermediate but also a versatile functional material in itself. This is due to the presence of 2-bromo-substituted thiophene and ω-hydroxyl functional end-groups, and due to the multiple functionalities encoded in its structure (photosensitivity, water self-dispersibility, self-assembling capacity). Thus, analysis of its behavior in solvents of different selectivities revealed that **Th-OMeOx** forms self-assembled structures (micelles or vesicles) by “direct dissolution”.Unexpectedly, by exciting the **Th-OMeOx** micelles formed in water with λ_abs_ of the OMeOx repeating units, the intensity of fluorescence emission varied in a concentration-dependent manner.These self-assembled structures showed excitation-dependent luminescence as well. Attributed to the clusteroluminescence phenomenon due to the aggregation and through space interactions of electron-rich groups in non-conjugated, non-aromatic OMeOx, this behavior certifies that polypeptides mimic the character of **Th-OMeOx** as a non-conventional intrinsic luminescent material.

## 1. Introduction

As modern materials are expected to have increasingly more sophisticated properties, polymers with complex topology and composition are in the forefront. A way to create such materials is by controlling the architecture and properties of their building blocks, allowing tuning of macromolecular shape, size and chemistry. This is nature-inspired fashion, through which complex functionalities of materials may be mediated [1].

Today, scientists can construct macromolecules of high complexity, with one possible technique being to endow conventional polymers with new functions suitable for a targeted application [2]. In this context, end-group functionalization of usual polymers [3,4,5], which evolved with the emergence and diversification of controlled polymerization methods tolerant to functional groups [6], allows for manipulation of the specific functionality of the end groups of the chains.

While the influence of well-defined polymer chain ends on physical properties has emerged as an important consideration for a number of applications [7,8], on the other hand, the high fidelity of chain-end functionality has changed chemists’ view of polymers from less-defined structures to versatile building blocks for the synthesis of more complex materials [4].

Thus, end-group functionalization of homopolymers with a polymerizable moiety it is certainly one of the most important milestones of polymer chemistry. It paves the way toward macromolecular architectural complexity, allowing the change from linear to nonlinear architecture through a so-called “macromonomer” or “grafting-through” strategy [9,10]. The turning point of interest for the synthesis and study of macromonomers was related to their need in non-aqueous dispersion (petroleum) polymerization (NAD) by ICI [9]. However, the main interest of a “macromonomer “ or “side-chain-first” strategy has been to obtain polymerizable precursors [10,11] for easy access to grafted (co)polymers [12]. Graft-shaped polymers are considered the most important polymer structure among the whole class of branched architecture [2].

Over the years, macromonomers [13,14] have steadily gained ground, subsequently leading to a new class of polymeric materials denoted polymacromonomers, (macro)molecular (bottle)brushes or “cylindrical polymer brushes” [10,15]. This new class is a collection of materials which, due to unique chemical design, show properties that are distinctly different from their linear analogues, allowing applications including electronics, optics, sensing [15] and various other biomedical fields [16,17,18].

Electroactive macromonomers appeared around the year 2000 [19,20], when a functional moiety having the capability to polymerize by electrochemical oxidative polycondensation [21], plus other methods for conjugated polymer (CP) synthesis [22,23], was placed as the end-group for various well-defined, flexible polymer chains [19,20,24,25,26,27,28,29,30,31,32,33,34,35,36,37,38,39,40,41]. 

These end-groups not only allowed the induction of properties that these flexible, commodity polymers never had [8,42] but greatly impacted the class of CPs, enabling the synthesis of grafted CPs (g-CPs) having well-defined oligomeric/polymeric side chains. Thus, the appearance of electroactive macromonomers afforded the movement of the so-called “solvent skin” concept of CP side groups beyond the solubility paradigm [43], while changing the classical amphiphilicity paradigm [44,45].

Besides that, g-CPs can be processed from solution by drop- [46] and spin-casting, ink-jet printing [33,47], electrospinning [27,48] and by 3D printing [31] in various form factors such as nanoparticles [36,38], thin films [33,45,46,47], nanofibers [27,48] or 3D constructions [29,31], with the oligomeric/polymeric side chains preserving [36,38,39] or enhancing [47] the properties of parent CPs. All of these aspects are beneficial when g-CPs applications are considered, in particular for optoelectronic devices. However, for good performance of such devices, g-CPs’ thin-film morphology is also important.

In this context, it is significant to notice that by polymerization of electroactive macromonomers, brunched copolymers of rod-*g*-coil type are obtained. Similar to linear rod-coil block copolymers, for g-CPs, the chemical and rigidity mismatch between the conjugated rod and flexible coils enable and impact phase separation and self-assembly. Thus, the presence of the anisotropic rod segment provides additional structural control and functionality, (including the π–π interaction and crystalline or liquid crystalline characteristics), creating orientational ordering as an important factor in the equilibrium behavior [49].

The key-role of the well-defined side chains in g-CPs, more than allowing low-cost manufacturing from solution, is related to a complex effect on the solution’s hierarchical self-assembling pathway during processing, offering large modulation of thin-film multiscale morphology, which dictates the ultimate device performance [50]. 

From this point of view, the architecture of g-CPs is an alternative “full of options” for many types of applications because it allows control of the processing–structure–properties relationship from the molecular design stage, when self-assembly can be programmed and structurally optimized [51,52].

Experimentally, several devices (based on various electroactive macromonomers-derived g-CPs) were constructed, for which the performances were adjusted and improved by changing side-chain number, regio-positioning, lengths or nature [25,33,53,54,55,56], illustrating the versatility of g-Cps as materials. In addition, side chains induce new properties to g-CPs, such as water-dispersibility, “stealth behavior” [36,38,41], thermo- and pH-sensitivity [30,34], intrinsic stretchability, self-healing properties [55,57], biocompatibility and partial biodegradability [28,29,31,46].

Some of these functionalizations facilitate the entrance of CPs into the field of biomaterials [58], synergistically fitting the goal of imparting electronic conductivity to biomaterials. In fact, the multitude of innate properties of CPs (for example photo-, electro- and electrochemical activity, light-harvesting capability and mechanical properties) and their general biomimicry [59] motivate the choosing of CPs as appropriate candidate materials for tissue engineering [60,61], imaging, diagnosis, various types of therapies [62] and biosensors [63]. 

In particular, from all classes of CPs, polythiophenes (PTh) are one of the most suitable for bioapplications [64,65,66]. More specifically, grafted PTh, (g-PTh) or its derivative g-PEDOT have been synthesized and tested for various fields. Thus, the inherent polythiophene conjugated main chain fluorescence in conjunction with biocompatible and functional side chains has been exploited for non-targeted [38] and targeted [67] cell imaging, targeted drug delivery and radiotherapy [68]. On the other hand, such materials were found to work as excellent biocompatible and electroactive cellular scaffolds [29,31,32,69,70,71], as well as active surfaces for selective adsorption of proteins [40]. Sensing of biomolecules (enzymes, neurotransmitters, cancer biomarkers) or illicit drugs is also possible using g-PTh or g-PEDOT [28,35,46,72].

As such, exploration and synthesis of new electroactive and photoactive thiophene macromonomers, derived from oligo/polymers already known for their biocompatibility and/or biodegradability and able to work as synthons in the obtainment of new biomaterials, is an interesting current topic [29,30,41,42].

In the context of synthetic polymeric biomaterials, poly(2 alkyl-2-oxazolines) (POXA) could be seen as a member of the “next generation”, having greater versatility and more diverse architectural possibilities to meet the new challenges and requirements of different biomedical applications [73], which range from delivery of various types therapeutics [73,74,75], tissue engineering [76] and antifouling and antimicrobial systems [77]. Hydrophilic poly(2-methyl-2-oxazoline)s (PMeOx) and poly(2-ethyl-2-oxazoline)s (PEtOx) are frequently compared to poly(ethylene glycol)s (PEG), not only because of the chemical similitude of the structural units, having a heteroatom separated by an ethylene bridge, but especially due to the demonstrated biocompatibility and stealth behavior toward plasmatic proteins [73]. Although out of all water-soluble polymers, PEG remains the gold standard in polymer-based biomedical applications, it has some drawbacks and limitations [78], including the noticed presence of anti-PEG antibodies in a significant number of healthy donors never treated with PEGylated therapeutics [79]. That is why research in academia and pharmaceutical companies is focused on finding alternative macromolecules for PEG replacements [78]. POXA has the potential to offer more than solely a substitute for PEG because it provides more highly versatile chemistry, allowing functionalization of both chain ends besides the side chains and modulation of the hydrophilic–lipophilic balance (HLB) of amphiphilic copolymers [18,80]. 

Surprisingly, in spite of the fact that both classes of polymers (CPs and POXA) are interesting and full of potential, to the best of our knowledge there are only a few papers in which conjugated polymers are described in conjunction with poly/oligo(alkyl-2-oxazolines) [81,82,83,84,85,86,87,88,89]. Most of the examples reported in the literature refer to amphiphilic g-CPs containing PMeOx of various lengths as side chains, but none of them contain the side chains directly connected to a thiophene ring.

As we found this aspect noteworthy, in the present paper we report the synthesis, structural characterization and property investigation of a new amphiphilic anisotropic compound based on a 2-methyl-2-oxazoline oligomer functionalized at its α-end with a photo- and chemically reactive thiophene end group. We obtained a material with interesting and relevant properties for bioapplication and with biomimetic behavior, as well as a reactive precursor for oligomers and amphiphilic g-CPs synthesis.

## 2. Results and Discussion

### 2.1. Design, Synthesis and Structural Characterization of Thiophene-Ended Oligo(2-Methyl-2-Oxazoline) Macromonomer (**Th-OMeOx**)

The criteria taken into account when the new macromonomer **Th-OMeOx** was designed are briefly illustrated in Scheme S1.

Thus, **Th-OMeOx** was projected based on moieties that form two contrasting parts. One of them is planar, aromatic, hydrophobic, low molecular weight, photosensitive, able to direct self-assembly by π–π stacking interaction with other aromatics and with polymerization capability. The second one, OMeOx, is oligomeric and aliphatic, able to coilup and is water soluble. Moreover, this part, which can be viewed as a conformational isomer of polypeptides, contains in its structural units a hydrogen bond acceptor group (-C=O), while the ω-end functional group is a hydrogen bond donor (-OH).

**Scheme 1:**
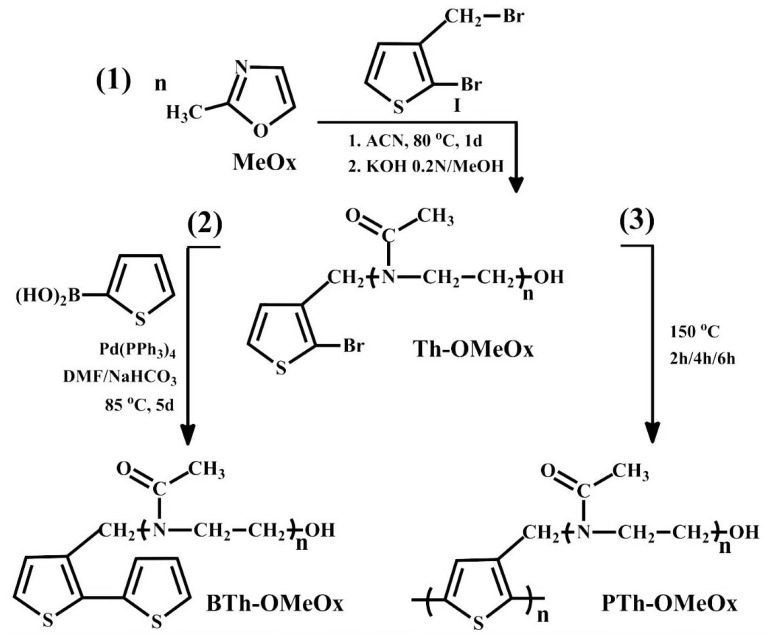
Synthesis pathway of macromonomer **Th-OMeOx** (**1**); synthesis of bithiophene macromonomer **BTh-OMeOx** obtained by Suzuki coupling (**2**); polymerization of macromonomer **Th-OMeOx** in solid state (**3**).

Using a commercially available thiophene derivative, (2-bromo-3-(bromomethyl)thiophene), that possess at the 3-position the -CH_2_Br functionality, usefully acting as electrophilic initiator for 2-methyl-2-oxazoline (**MeOx**) cationic ring opening polymerization (CROP), the new macromonomer was synthesized as depicted in Scheme 1 [81,84]. 

As the polymerization reaction was quenched by the addition of KOH methanolic solution, the placing of the hydroxyl functionality at the opposite end of the OMeOx obtained chains was possible. Besides having geometric shape, electronic character and dissymmetrical solvophilicity character, **Th-OMeOx** contains one reactive bromine attached at the 2-position of the thiophene ring, which additionally enhances its amphiphilic balance. Based on this design, in solutions of selective solvents, it could be possible to make use of hydroxyl end-functional groups to drive hydrogen-bonding-induced intermolecular π–π stacking of **Th-OMeOx** molecules for photophysical property modulation [90].

The basic structural characterization of macromonomer **Th-OMeOx** was performed by NMR and FTIR spectroscopy. Before describing the results obtained by NMR, some important clarifications need to be added regarding the structure of the macromonomer and how it affects these results. As given in a detailed manner in Scheme S1, **Th-OMeOx** was designed based on the characteristic criteria of “shape amphiphiles” [49]. Thus, it not only shows solvophilic amphiphilicity due to the hydrophobic and hydrophilic constitutive parts, but its structure is characterized by the shape and stiffness anisotropy. The shape effect and the mismatch in rigidity of **Th-OMeOx** and also its **BTh-OMeOx** derivative (Scheme 1) are overwhelming factors that lead to the self-assembly of these “block molecules”, which behave in solution as block copolymers do.

Under these conditions, it is expected that the results of NMR spectroscopy will be influenced by polydispersity and the short length of the OMeOx chain, but, equally important, by the formation of micellar structures by self-assembly. All of these influences, just like in the case of block copolymers, affect the intensity, the number and the positions of signals in both ^1^H- and ^13^C-NMR spectra (see more details in Supplementary Material).

**Figure 1:**
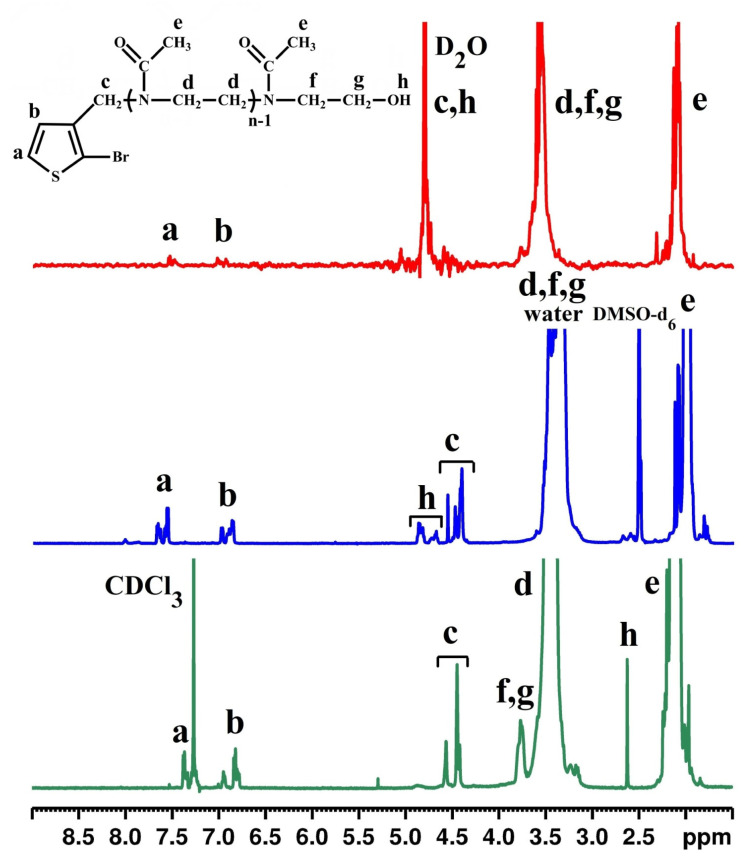
The ^1^H-NMR spectra of **Th-OMeOx** macromonomer in various solvents.

In order to prove as accurately as possible the obtainment of the new macromonomer, the ^1^H-NMR spectra were registered in three different solvents (Figure 1). Such an approach avoided superposition of some signals from the compound with those of the solvents used for the registrations, in spite of changing the registration solvents selectivity in relation with the constitutive parts of **Th-OMeOx**. Because in water and in chloroform **Th-OMeOx** forms self-assembled micelles (as will be proven later by other investigations), their presence could affect the ^1^H-NMR results.

In the spectrum registered in CDCl_3_ it was possible to exactly identify and assign the peaks for aliphatic methyl (**e**) and methylene (**d**, **f** and **g**) protons from OMeOx, and also proton **h** from the hydroxyl functional end group of the oligomer. This type of proton was also identified in the spectrum registered in DMSO-d_6_, but shifted downfield and as two groups of broad, split peaks. Such behavior could not only be due to the registration solvent’s increased polarity and its favorable interaction with the hydroxyl proton, but also due to micellization, enhanced by the water inherently present as an impurity in DMSO-d_6_.

If the signal of protons **a** and **b** of the thienyl moiety appeared superimposed with that of the non-deuterated traces of chloroform (Chl) in CDCl_3_, in the spectrum registered in DMSO-d_6_, these signals appeared alone in the region of 7.4–7.6 ppm **a** and 6.8–7 ppm **b**, respectively. In the spectrum registered in D_2_O, a significantly decrease can be noticed in peak intensity of aromatic protons **a** and **b**. This is a consequence of **Th-OMeOx**’s amphiphilic character and concomitantly evidence (besides the other evidence described in the next subsection) of its micellization by self-assembling in water as a selective solvent for the OMeOx part. 

It is also important to point out that in any of the used solvents, correlation between proton intensity valid for the ideal situation cannot be possible in our spectra. This is due to the fact that it is hard to find a solvent that works well for both parts of the **Th-OMeOx** molecule due to its specific structural characteristics.

In this respect, based on observation of the signal of methylene proton **c**, placed in the vicinity of the aromatic thienyl ring and originating from the initiator, it can be stated that it is the most sensitive to modification due to the solvents used for spectral recording. If in D_2_O this proton is supposed to appear together with those of the non-deuterated water traces and with proton **h** belonging to the final hydroxyl groups, in CDCl_3_ and DMSO-d_6_ the results are far from the expected ideal singlet. Thus, in CDCl_3_, the split peak from 4.45 ppm and the small peak from 4.56 ppm were both assigned to the type **c** protons; in DMSO-d_6_, the peak group from 4.4–4.55 ppm was assigned to the same(**c**) protons. It was assumed that the position and shape of these signals, which deviate from the theoretically expected ones, could be due to the sensitivity of proton **c** to the variation in length of the short OMeOx (polymerization degree (PD) = 18) directly connected to the carbon atom in the third position of the thiophene ring, even if a low value was found by GPC for index of polydispersity (IPD) (vide infra). In other words, both the position and the shape of the signals assigned to this type of protons are sensitive to OMeOx polydispersity. On the other hand, because OMeOx chains are short, the chance of the appearance of signals characterizing **Th-OMeOx** having attached OMeOx of different lengths is high. In fact, these experimental findings in both solvents suggest that the CH_2_ group connecting the thiophene ring and OMeOx is placed in more than one chemical environment due to a combination of factors, such as solvent, OMeOx’s polydispersity and micelles forming by self-assembly (see Supplementary Material for additional details).

**Figure 2:**
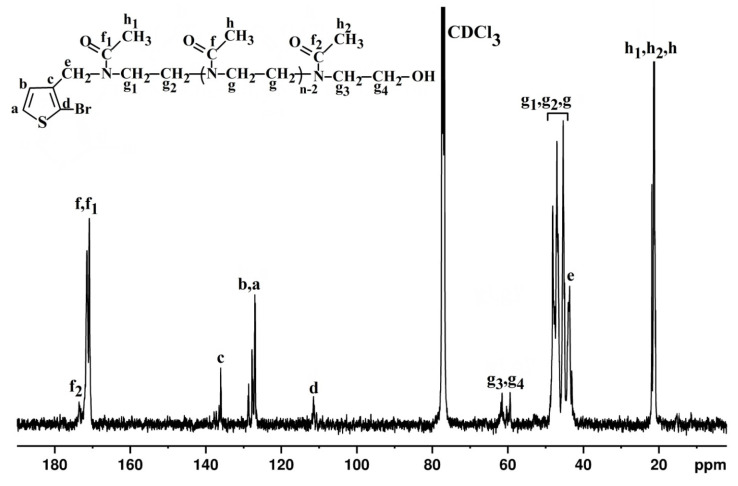
The ^13^C-NMR spectrum of **Th-OMeOx** macromonomer registered in CDCl_3_.

In order to complete the structural information given by ^1^H-NMR, the ^13^C-NMR spectrum of **Th-OMeOx** in CDCl_3_ was registered as well (Figure 2). It is worthy noting that not only in the ^1^H-NMR spectrum but also in ^13^C-NMR, the signals of the atoms from the repeating unit placed at ω-chain-ends appeared separately, supporting that the hydrophilic part of the macromonomer is oligomeric.

Moreover, ^13^C-NMR (Figure 2) revealed, as separate signals, the carbon atoms from the first repeating unit in the immediate neighborhood of the aromatic thiophene ring. In accordance with this feature, three signals attributed to the carbon atoms in the carbonyl moieties appeared in the range 170.83–171.47 ppm (**f** and **f_1_**) and at 173 ppm (**f_2_**). Further, the signals specific to carbon atoms of the three types of methyl groups (**h**, **h_1_**, **h_2_**) can be seen in the range 20–22.3 ppm. The peaks in the range 44.8–48.7 ppm were assigned to the carbon atoms of methylene (**g**, **g_1_** and **g_2_**) in OMeOx repeating units. As expected, the carbons of the methylene moieties from the repeating units at OMeOx ω-chain-ends appeared in a separate region as **g_3_** and **g_4_**. Peaks denoted as **e** (43.7 ppm) in the methylene group and **a** (126.99 ppm), **b** (127.06 ppm), **c** (136.03 ppm) and **d** (111.48 ppm) of the aromatic thiophene ring originating from the initiator are present as well, but as a complicated shape. This may correlate with the above described situation of protons **c** in the ^1^H-NMR spectrum, which are originating from the initiator. So the influence of the short length of OMeOx and of its polydispersity could be reasons for the complicated signal appearance in the^13^C-NMR spectrum.

Even if the NMR signal intensities are affected by self-assembly, calculation to estimate the degree of polymerization for OMeOx was performed from the ^1^H-NMR spectrum registered in DMSO-d_6_ due to the fact that the peaks of protons **a** and **b** of the thienyl end groups were not affected by the signal of the solvent. 

Thus, the intensity of peaks **a** and **b**, attributed to thiophene, was compared with that of peak **e** or **d**, and a polymerization degree (PD) of 18 was obtained, corresponding to a molecular weight of macromonomer **Th-OMeOx** of M_n, H-NMR_ = 1723. 

Based on the data presented above, in the case of the **Th-OMeOx** macromonomer, structural characterization by NMR must be accompanied by other complementary data that, in a convergent way, supports the structural characterization. Thus, to add more information, the infrared spectrum of the synthesized compound was registered and is given in Figure S1. It shows the characteristic vibrations of both the OMeOx chain and the thienyl ring as follows: 3449 cm^−1^OH from OMeOx chains ends; 3080 cm^−1^ C–H stretching vibrations of thienyl ring; 1637 cm^−1^C=O carbonyl stretching from OMeOx; 1420 cm^−1^ deformation vibration of CH from CH_3_ of OMeOx; 1255 cm^−1^ stretching and bending vibrations (υ C–N, υ C–C and δ C–C) of OMeOx backbone [81]. Further, signals attributed to the 2,3-disubstituted thiophene moiety were identified at 1364cm^−1^ (υ_ring_), 1012 cm^−1^ (υ C-Br), 925 and 826 cm^−1^ (β CH), 764 cm^−1^ (δ CH) and 726 cm^−1^(-C-Br). The absorbance at 609 cm^−1^ and 502 cm^−1^ should be attributed to C–S bending and C–S–C ring deformation, respectively [38,46].

UV–vis spectroscopy added new information to confirm the obtainment of the **Th-OMeOx** macromonomer. Thus, Figure S2A shows the absorption traces for initiator 2-bromo-3-(bromomethyl) thiophene and for the obtained macromonomer registered in acetonitrile. It can be seen that, while the initiator shows two absorption maxima (213 nm and 245 nm), for the macromonomer, three peaks are present: the first one, more intense and blue-shifted at 201 nm, the second one at 245 nm of a decreased intensity, and also a small and shallow one at 322 nm. The first two absorptions are due to the thiophene ring in the **Th-OMeOx** structure, while the one at 322 nm, of lower intensity, might be attributed to absorption by the carbonyl groups in OMeOx structural units, as previously reported [91].

The apparent molecular weight of **Th-OMeOx** was also evaluated by GPC measurements in Chl and in N’-dimethylformamide (DMF) as solvents (Figure S3). Different molecular weights were obtained, most probably due to OMeOx’s different conformations in the two solvents used for registration (collapsed in Chl as a marginal solvent for OMeOx and extended in DMF as a good solvent). Further, discrepancy between the polar, hydrophilic nature of OMeOx in comparison with the non-polar, hydrophobic polystyrene used as the standard in the GPC columns could influence the obtained values. **Th-OMeOx** has a propensity for self-assembling (SA) in selective solvents, including DMF and Chl, and supposedly formation of self-assembled structures with different sizes and shapes can decisively impact GPC results. However, the GPC traces in both solvents were unimodal and narrow, indicating that no side reaction occurred, and that polymerizations took place in a controlled manner. Compared to the molecular weight obtained by ^1^H-NMR, the value of 2787 (index of polydispersity, IPD = 1.03) obtained by GPC in DMF is higher, while that of 1418 (IPD = 1.14) obtained in Chl is lower (see Figure S3 in Supplementary Materials).

### 2.2. Properties of **Th-OMeOx** Macromonomer in Different Solvents and in Thin Films

Constructed as an amphiphilic, π–aromatic group-ended system, as in the case of previous kindred structures [42], **Th-OMeOx** is expected to undergo SA in selective solvents, forming supramolecular structures [92,93]. Such a phenomenon, which can generate complexity at the nanoscale, can have implications on the properties both in solution and in films, and can influence **Th-OMeOx**’s biological interactions.

As such, the impact of structural details and the influence of solvent polarity or concentration on **Th-OMeOx** SA and on the morphology in thin films have been investigated by using a combination of spectrophotometric techniques (UV–vis and fluorescence spectroscopy) with dynamic light scattering (DLS) measurements and with atom force microscopy (AFM).

Taking advantage of the thiophene ring at the α-end of **Th-OMeOx**, its photophysical properties were scrutinized in solvents, showing particular selectivity in relation to the constitutive elements of its amphiphilic structure (see Table S1 for solvent characteristics). The sensitivity of fluorescence emissions, which differentiates the variations that occur during SA, has been exploited to obtain more information on weak, intra- and intermolecular forces that govern **Th-OMeOx**’s behavior in the chosen solvents. 

Thus, water was taken as selective for OMeOx, Chl as a marginal solvent for OMeOX [94] (but selective for bromo-substituted thiophene ring), while tetrahydrofuran (THF) was considered as a non-selective polar solvent (or rather as slightly selective for OMeOx, if the values of the components of Hansen solubility in Table S1 are compared).

To investigate how variation of concentration of colloidal dispersions influences **Th-OMeOx**‘s relevant properties, such as the size of self-assembled micellar nanoparticles or their photophysical properties, experiments modifying this parameter were performed in water, as a relevant medium for bioapplications.

**Figure 3:**
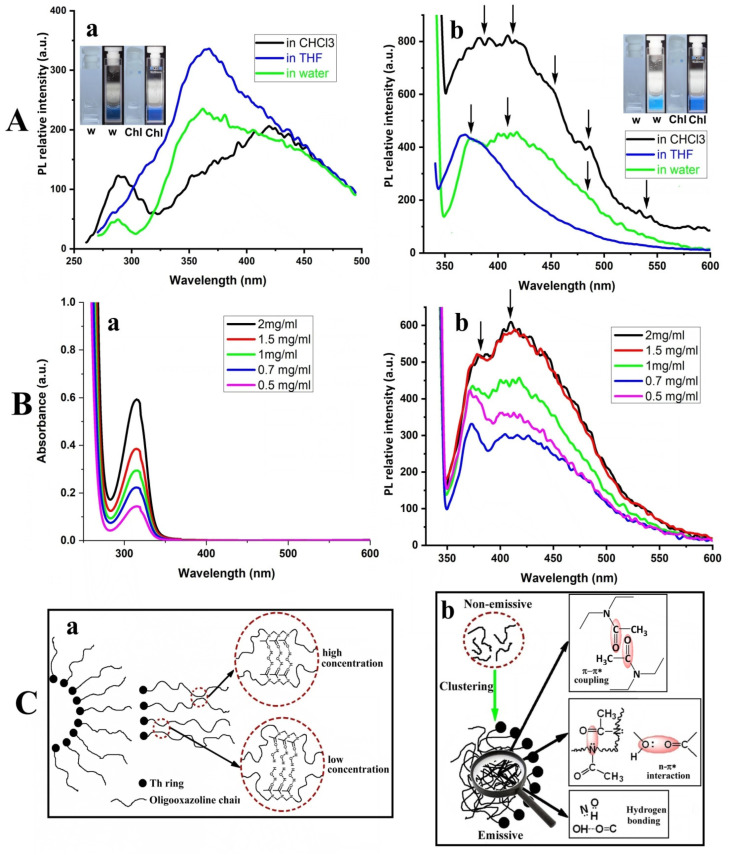
(**A**) Fluorescence spectra of **Th-OMeOx** in different solvents at λ_ex_ = 260 nm (**a**) and at λ_ex_ = 330 nm (**b**); (**B**) UV–vis spectra (**a**) and fluorescence spectra (**b**) of **Th-OMeOx** registered in water (c = 1 mg/mL) at different concentrations, using λ_ex_ = 330 nm for fluorescence; (**C**) schematic representation of presumptive behavior of OMeOx chains in SA micelles formed in water at low and high concentrations (**a**) and in SA micelles in Chl (**b**); the inset in (**A**) shows photos of dispersion in water (w) and in Chl in daylight and under UV light (256 nm (**Aa**) and 365 nm (**Ab**)).

Some of the experimental data obtained by DLS, fluorescence and UV–vis spectroscopy are collected in Table 1 and Table S2 and Figure 3, Figure S2 and Figure S4, respectively.

**Figure 4:**
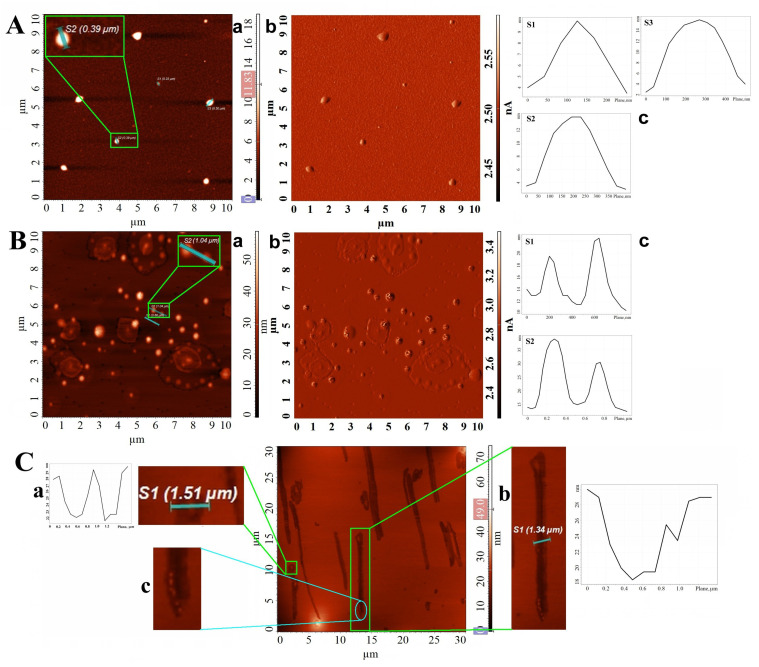
(**A**) AFM height (**a**) and amplitude (**b**) image of the round-shaped particles formed by drop-casting **Th-OMeOx** dispersion in water; (**c**) cross-sectional analysis of the particles shown in magnified insets in (**a**); (**B**) AFM height (**a**) and amplitude (**b**) image of the round-shaped particles formed by drop-casting **Th-OMeOx** dispersion in Chl; (**c**) cross-sectional analysis of the particles shown in magnified insets in (**a**); (**C**) AFM height image (middle panel) of self-assembled structures formed by drop-casting **Th-OMeOx** dispersion in THF; magnified height image and cross-sectional analysis of (**a**) round-shaped vesicle and (**b**) rod-shaped vesicle; (**c**) magnified area (4×) showing fusion of round-shaped small vesicles at the bottom of a rod-shaped vesicle.

DLS measurements (Table 1 and Figure S4) prove that **Th-OMeOx**, by simple “direct dissolution”, forms self-assembled supramolecular structures in all investigated solvents. Their sizes are solvent- and concentration-dependent and the AFM investigation showed that formed particles, of micellar type both in water and in Chl, are round in shape (Figure 4A,B). For these two solvents, the apparent hydrodynamic diameter (D_h_) in dispersion and also the size in the dry state ranges from 250–520 nm (Table 1, Figure 4A,B).

As can be seen from the data in Table 1, in water, the D_h_ of micelles increased with increasing dispersion concentration. The only noted exception is for the value of c = 0.5 mg/mL, when the registered value of D_h_ was the highest. This is most likely due to the low thermodynamic stability of the first-formed structures, which subsequently associated to form larger ones for increased colloidal stability.

When Chl was used as solvent, selective for the 2-Br-Th moiety (Table S1), also the value of D_h_ was high at this concentration. As in this situation the shells of micelles consist of substituted thiophene rings of small dimension, a higher number of **Th-OMeOx** molecules are necessary to associate to attain colloidal stability. For THF, the DLS trace (Figure S4) is trimodal, advocating a mixed population of self-assembled objects.

However, due to DLS limitations, the obtained results, in particular in THF, should be considered with care, as the technique is based on the assumption that the particles are non-interacting spheres and thus eventually the presence of anisotropic structures with another shape could induce errors.

More insight into the SA came from analyzing the results of the fluorescence measurements (Figure 3). When the samples in the three solvents were excited with λ = 260 nm, (a value around the λ_max_^abs^ of the thiophene ring, as given in Table S2), a difference in shape, intensity and position of emission peaks was noticed between water and Chl compared to THF (Figure 3Aa). This suggests distinctly different SA behavior in THF than in both other cases. Not only the position and the values of the intensities of the appeared emission peaks maxima, but also their number are different. Moreover, there is not a systematic relationship between the observed differences and the variation of the polarity of the solvents (Table S1). Thus, this observation supports the conclusion that the different experimental results of the fluorescence measurements are due to the **Th-OMeOx** self-assembly, which went differently in the three solvents due to their different selectivity. In addition, a different type of supramolecular self-assembled structure in THF is suggested by the fluorescence trace in Figure 3Aa, which was subsequently confirmed by AFM (Figure 4C).

It was also interesting to notice that the photoluminescence spectra of the self-assembled structures in water and in Chl, when excited with 260 nm, showed dual emission peaks (Figure 3Aa, Table S2). The first emission maxima (288 nm for water and 291 nm for Chl) might be due to the associated thiophene rings, located in the confined spaces of the core or of the shell of the formed micelles, depending on the solvent. 

It seems that the placement of the self-assembled, dissimilar constitutive parts of **Th-OMeOx** in separated but confined spaces of the formed supramolecular structures is the key aspect for the fluorescence measurement results. Such a claim can be supported by the fact that, as can be seen in Figure S6A, when the ACN solution of the initiator was excited with a wavelength of 260 nm, no emission was registered. In THF, a shoulder of the only peak is discernible at 284 nm, placed before its maximum at 366 nm (Figure 3Aa). 

For water and Chl, the second emission peak, with a higher intensity than the first one, has a broad spectral width (approximately 200 nm), covering almost the whole blue region. The same broadness is shared also by the unique peak from THF (Figure 3Aa). 

Such emissions in the blue region also appeared when the dispersions in all solvents were excited with 315 nm (Figure S5D) and 330 nm (Figure 3Bb) wavelengths, which are in the regions attributable to λ_abs_ of OMeOx (vide supra). Thus, it can be seen from Table 1 and Figure 3Ab that the emission maxima are red-shifted with the increasing of λ_ex_ in all the investigated solvents.

Further, the emission peaks changed their intensity in addition to the general emission profiles changing (Figure 3Ab). This excitation-dependent fluorescence could indicate the presence of various stable excitation states with different energy levels. Moreover, the appearance of multiple emission peaks can be noticed at the unique λ_ex_, in particular in Chl (marked with arrows in Figure 3Ab), that might be assigned to different emitting species existing in the self-assembled structures of **Th-OMeOx** in this solvent.

All of these results highlight a behavior which is uncommon for a compound containing an oligomer of non-aromatic and non-π-conjugated nature in 90% by weight. Moreover, although OMeOx lacks any π-conjugated fragment, **Th-OMeOx** produces bright blue emission under UV light, both in water and in Chl, as shown in the insets in Figure 3A.

Furthermore, it is important to notice that the emission in the blue region is only present for self-assembled **Th-OMeOx**, and it is missing for bare, non-associated, commercially available PMeOx (PMeOx, c.a.) (Figure S4D and Figure S5 and additional discussion in Supplementary Material).

In this regard, it should be emphasized that in the last decade considerable interest has been directed towards the development of light-emitting materials, including a variety of synthetic or natural polymers having non-aromatic heteroatom-based structures devoid of adjacent π-conjugated sequences, but with a saturated σ bond that separates functional groups (such as –OH; –NH; –NH_2_; –COOH; –O–C=O; –O–; NH–CO–;) bearing π and/or lone pair (n) electrons [95]. They generally show an unusual [96] “mysterious” and “magic” intrinsic blue luminescence [97] in their aggregated state and/or under physico–chemical confinement [98]. Emerging concepts of “clusteroluminescence” (CL) [98] and “clusterization-triggered emission” (CTE) [99] were proposed for the mechanism of this atypical luminescence, which is still under debate [100]. These materials show luminescence only while clustered, being non-emissive in a molecularly dissolved state or in dilute solutions. Their emissive properties cannot be explained by the traditional photophysical theory of “through-bond conjugation”, and the CTE mechanism implies “through-space interactions” and “through-space conjugation” mediated by non-bonding interactions of the electron-rich moieties; the resulting extended electron delocalization together with concomitant conformation rigidification are conducive to efficient luminescence [101]. Besides the characteristic molecular structure, these polymers also share certain common photophysical features such as: (i) excitation-dependent luminescence; (ii) concentration-enhanced emission; (iii) size-dependent emission properties that are enhanced with increased size (e.g., molecular weight, dendrimer generation or nanoparticles size); (iv) multiple emission peaks when λ_ex_ is near the value of λ_abs_; (vi) room temperature phosphorescence [102].

When a system works as a CTE, its typical effect can be directly observed by comparing the fluorescence intensity at different concentrations [99]. In this regard, if the experimental results in Figure 3B are taken into account, it can be seen that the intensities of both absorption and fluorescence emission in water increased with increasing **Th-OMeOx** concentration. Moreover, dependence of emission intensity on the size of the micelles can also be highlighted in Figure 3Bb and in Figure S5D. Thus, the emission intensity at the concentration of c = 0.5 mg/mL is higher than that at 0.7 mg/mL according to the size of the micelles listed in Table 1. This variation emphasizes once again the increased sensitivity of fluorescence compared to other properties of emitting nano-objects. In Figure 3Bb, two emission maxima (marked by arrows) are seen in all the traces. It is interesting to notice that by increasing the dispersion concentration, the ratio of the intensities of these maxima changes, as the higher position’s intensity increases as those at the lower wavelength decrease. Such a phenomenon was already reported for polymers containing a carbonyl group in different types of functionalities, evidencing multifarious packing structures existing in clusteroluminogenes [103,104].

It seems that for **Th-OMeOx,** the first appearing emission maximum at the shorter wavelength stems from the carbonyls of the pendant acyl groups, which are predominantly water-dispersed along the OMeOx chains as individuals, being in a higher amount at the lower concentrations (see the sketch in Figure 3Ca); most probable they are not easily excitable. This supposition can be supported by the result of fluorescence measurement of PMeOx c.a., in the trace of which only the peak at 357 nm is present (Figure S5D and additional discussion in Supplementary Material). The origin of the emission peak at the higher position might tentatively be attributed to the locked tertiary amide functionality due to enhanced interchain interactions and clustering inside the micelle shell at high concentrations.

As represented in the sketch in Figure 3Ca, at higher concentrations water molecules (in a lower ratio per repeating units) could act as mediators, linking two neighboring chains via dipole interactions and hydrogen bonding, an already documented phenomenon [105,106]. Thus, the appearance of the second emission peak and of the blue luminescence of **Th-OMeOx** in water at high concentrations could be mainly due to the formation of chromophoric sequences with effective through-space electronic communications, with extended delocalization and rigidified conformations, as shown in the circle area in Figure 3Ca, while multiple hydrogen bonds suppress the non-radiative process. Thus, the emission phenomenon is a result of “through-space interaction” and the hydrogen bond-induced clustering of the pendant carbonyl groups. If the importance of water molecules bound to proteins on emissions is taken into account [107], then it can be emphasized that **Th-OMeOx** behaves in a biomimetic way in water dispersion.

When **Th-OMeOx** is dispersed in Chl at the same concentrations as in water, the blue luminescence under UV irradiation is also evident, seemingly with higher brightness (Figure 3A). Furthermore, with increasing the excitation wavelength, the emission maxima were found to be red-shifted, accompanied by emission intensity enhancement (Table 1, Figure 3A and Table S2). The difference in fluorescence traces’ shape compared to those in water suggests an accordingly different emission mechanism. The first obvious reason is the difference in OMeOx chains placement—this time in the confined space of the micelle core. In this case, due to solvent selectivity and properties (Table S1) but also due to confinement, OMeOx chains are collapsed. In this scenario, clustering is favored, and possible intra- and intermolecular interactions and conjugations “through-space” have led to π–π* couplings, n–π* interactions and the formation of hydrogen bonds between π and/or lone pair (n) electrons contained in the tertiary amide functions and the hydroxyl terminal ω end-groups of OMeOx (as schematically described in Figure 3Cb). All of these could synergistically contribute to delocalization of the electrons and to the rigid conformation. Thus, in Chl, emission comes from the concomitant existence of collapsed conformation and the clustering of OMeOx chains into a confined space, which enables various stable excited states with many different energy levels.

Taken together, the results of the photophysical investigations reveal that OMeOx in water or in an organic non-polar solvent, in an extended, rigidified conformation or in a collapsed one behaves as a non-conventional luminophore, with blue emission into the frame of the self-assembled structures formed by the amphiphilic **Th-OMeOx** compound. Obviously, there are still many issues to be clarified in this regard, requiring in-depth studies, but which go beyond the scope of this paper. One of them is the possible impact of the 2-Br-Th α-end group on OMeOx’s blue emission apart from its hydrophobic character, aromaticity and the shape it endows **Th-OMeOx**, with complex amphiphilicity.

For example, the self-assembled thiophene rings (inside the core of water micelles or in the shell of micelles in Chl) could act as an energy donor in a FRET-type process when the dispersions were excited with a λ_ex_ value shorter than that of the λ_abs_ of OMeOx (Figure 3Aa), similar to π-conjugated “classical” systems [45]? The answer to this question and others regarding the role of the α-end 2-Br-Th group are very important, especially since there is only one report in the literature on the blue emission of OMeOx [108]. The authors stated that the origin of oligo(2-methyl-2-oxazoline) fluorescence must be related to the applied polymerization route using supercritical carbon dioxide, and to obtained carbamic acid α-chain ends, “since there are no reports on fluorescent poly(2-alkyl-2-oxazoline)s prepared by conventional methodologies” [108]. 

The general dependence of the properties on the shape and the size of the self-assembled structures is also supported by our experimental findings in THF. The results of the fluorescence measurements already suggested the formation of a different type of SA structure (namely a different molecular arrangement and mutual positions between **Th-OMeOx** structural components) compared to water and Chl (vide supra).

Indeed, AFM revealed the presence of vesicles having different shapes (Figure 4Ca,b) in films obtained by drop-casting the dispersion in THF. This was confirmed by cross-sectional analysis, emphasizing the shape typical of self-assembled bilayer structures (Figure 4Ca,b). These results were both unexpected and intriguing. It is important to remember that film forming is a dynamic process, and clear differentiation has to be made between the SA in solution and that on surfaces. This is because when a surface comes into play, additional forces and effects become predominant during solvent evaporation, which might lead to a completely different mode of SA.

The formation of self-assembled structures at the investigated concentration is supported by the DLS measurements (Figure S4C). Given the amphiphilic nature of **Th-OMeOx**, as well as its ability to behave similarly to block copolymers [109], the presence of such bilayer structures already in dispersion cannot be ruled out. 

However, as suggested by Figure 4Cc, those with a tubular shape, tens of micrometers long (Figure 4Cb), were most likely formed during evaporation of THF in the drying procedure (see Experimental Procedures) by the fusion of ovoid/round vesicles that also populate the investigated area (Figure 4C, middle panel). Explanation for this morphology may be found in THF being considered a selective solvent for OMeOx. In this hypothesis, a self-assembled aromatic 2-Br-Th layer, “embedded” between two layers of hydrophilic OMeOx, forms vesicular structures that contain a certain amount of solvent inside. After deposition on the mica support, during evaporation of the solvent and film formation, a change in the shape of the self-assembled structures formed in dispersion may occur. Thus, it is very probable that the affinity of the mica hydrophilic surface to the OMeOx shell (acting as a bonding force) and the possible presence of atmospheric water vapor facilitated the fusion in Figure 4Cc and the shape change.

All in all, the study of **Th-OMeOx** in different solvents shows that due to its peculiar structural construction it works as a multifunctional material with the capability to self-assemble in aqueous or organic media, forming nanostructured particles (micelles or vesicles) that behave like clusteroluminogens, showing blue luminescence under a wide range of excitation wavelengths, while in thin film of THF, surprisingly, tubular vesicles of tens of nanometers in length are formed, most probably during film formation with contribution from the support surface’s hydrophilicity.

### 2.3. Examples of Subsequent Modifications of **ThOMeOx**

The presence of bromine functionality in the structure of **Th-OMeOx** allows subsequent modification. The new reactions were performed in both solution and solid state as is shown in the following.

#### 2.3.1. Synthesis of 2,2’-3-OMeOx-Substituted Bithiophene Macromonomer (**BTh-OMeOx**) by Suzuki Condensation

**Figure 5:**
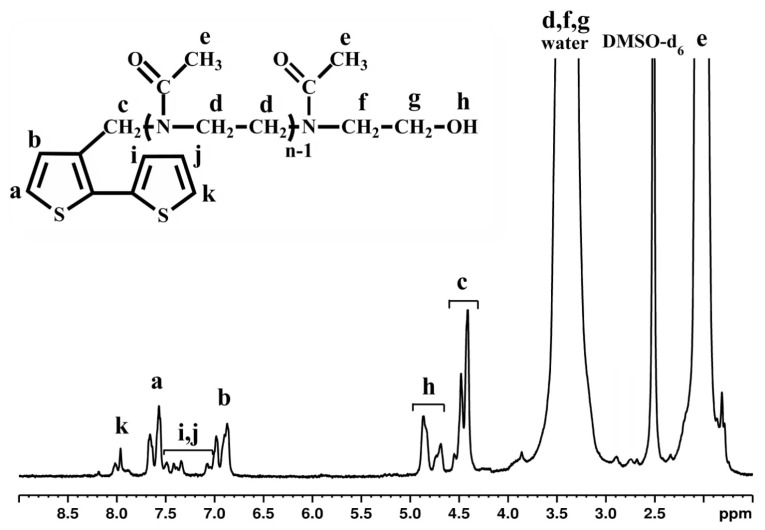
^1^H-NMR spectrum of macromonomer **BTh-OMeOx** in DMSO-d_6_.

As presented in Scheme 1, by applying Suzuki condensation in DMF, a bithiophene macromonomer having appended OMeOx at the third position of the first ring was obtained. Formation of the expected structure was proven by ^1^H-NMR (Figure 5) and FT-IR spectroscopy (Figure S1). 

By comparing with the spectrum of **Th-OMeOx**, the FT-IR spectrum of **BTh-OMeOx** shows several changes in the region which characterizes the aromatics, proving that the coupling reaction between the two thiophene rings took place. Thus, the peaks at 1012 cm^−1^ and at 726 cm^−1^ attributed to the C–Br linkage disappeared in the spectrum of **BTh-OMeOx**, a fact that supports bithiophene sequence formation. Further, the appearance of new peaks due to the presence of the thiophene ring in the bithiophene moiety can be identified at 952 cm^−1^, 837 cm^−1^ (β CH) and a broad peak centered at 585 cm^−1^ (γ ring deformation). Besides these, a group of five peaks (1509 cm^−1^, 1521 cm^−1^, 1540 cm^−1^, 1559 cm^−1^and 1574 cm^−1^) that can be attributed to the newly appearing conjugated sequence of bithiophe can be deciphered in the spectrum in Figure S1. 

Moreover, due to increased hydrophobicity through the addition of the second thiophene ring to **BTh-OMeOx**, and thus the HLB decreasing, the amount of retained water is expected to decrease as well. Shifting of amide carbonyl stretching from 1637 cm^−1^ to the higher wavenumber of 1648 cm^−1^ in the IR spectrum of **BTh-OMeOx** (Figure S1) suggests a decreased amount of associated water, which usually is related to the highly hydrophilic character of OMeOx [110]. This shifting correlates well with the results of thermogravimetrical analysis (TGA) (Figure S6), which revealed that the weight loss up to 100 °C that is attributable to the loss of physiosorbed water is only 1.41%, compared to 4.6% in **Th-OMeOx**. The addition of the second thiophene ring is also responsible for enhanced thermal stability of **BTh-OMeOx** in comparison with the starting **Th-OMeOx** if the initial degradation temperature (IDT) values are taken into account (343 °C and 239 °C, respectively; Figure S6).

^1^H-NMR spectroscopy verified the anticipated structure, and all the observable signals can be assigned to the respective protons of the structure, as shown in Figure 5. When comparing with the spectrum of **Th-OMeOx** registered in the same solvent (Figure 1), the differences are obvious, especially in the aromatic region of the spectrum, where the signals characteristic to the second thiophene ring (**i**, **j** and **k**) appeared in the range 7–7.5 ppm and 7.9 ppm, respectively. 

GPC measurement performed in Chl (Figure S3C) give a value of M_nGPC_ = 1939, higher that of the starting **Th-OMeOx**, as expected, with the IPD value being practically unchanged (1.11 for **BTh-OMeOx** in comparison with 1.14 for **Th-OMeOx**). These data confirm not only the obtainment of the new macromonomer but also that the integrity of the oligomeric 2-methyl-2 oxazoline was not affected during the Suzuki reaction.

Photophysical properties investigation, performed in the same solvents and at the same concentration (c = 1 mg/mL) as for the starting **Th-OMeOx**, also support the obtainment of the new bithiophenic structure. Generally, a red shift of both λ_abs_ and the maxima of λ_em_ can be noticed (Figure 3Ab and Figure S2B–D, Table S2). This trend is attributable to the newly formed bithiophene sequence. It is important to point out that bare 2,2′-bithiophene shows two absorption maxima at 250 nm and at 303 nm [111], while for its 3,3′-substituted derivatives, blue shifted values at 245 and 267 nm (sh) were reported [112]. As can be seen in Figure S2C, due to the used solvent’s cut-off, only in Chl are the two absorption peaks discernible (at 249 nm and 264 nm (sh)) that are assigned to the π–π* transitions of the thiophene ring and the bithiophene moiety, respectively. In all three solvents, absorption maxima placed after 300 nm can be seen as well (see Table S2 for the exact values), which are assignable to the carbonyl groups in OMeOx, in a similar manner as for **Th-OMeOx**.

In addition, the net difference between the shape, intensity and values of the maximum λ_em_ of the fluorescence traces of **BTh-OMeOx** in Figure S2D (λ_ex_ = 330 nm) compared to those of **Th-OMeOx** from Figure 3Ab is clear evidence of the obtainment of the new bithiophenic macromonomer.

**Figure 6:**
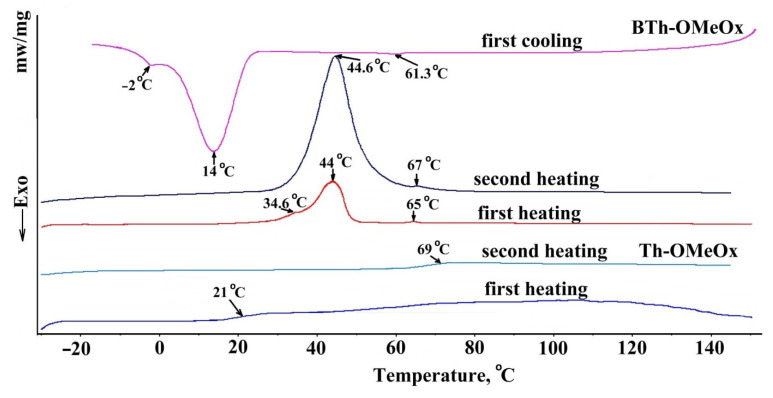
DSC traces of **Th-OMeOx** and of **BTh-OMeOx**.

In supporting this claim, evidence can also be gained from thermal property investigation. As was reported, the glass transition temperature (T_g_) of PMeOx is 82 °C [94] or 72 °C [113]. At a heating rate of 40 °C/min and in the temperature range of 30–130 °C, **Th-OMeOx** had a T_g_ of 65 °C in the first heating run of the compound, after the work-up during synthesis and purification (Figure S5B). When measured at the same rate but over an enlarged range from −20–140 °C (Figure 6, **Th-OMeOx** first run), surprisingly, the T_g_ appeared at a lower value of only 21 °C, accompanied by a very shallow endothermic with the maximum around 100 °C, which is most probably due to elimination of retained water. In the second run, after cooling, the T_g_ value reached 69 °C. This change reveals the influence of the recording conditions on the measured thermal parameters, while the difference of T_g_ values compared to those reported in the literature is likely the result of the difference between the molecular weights. As can be seen from Figure 6, the DSC traces of **BTh-OMeOx** are more complex compared to **Th-OMeOx**. Thus, in the first heating run of **BTh-OMeOx** after synthesis, T_g_ of OMeOxwas 65 °C. Interestingly, an asymmetric endothermic peak also appeared, with a main endotherm at 44 °C and a shoulder at 34.6 °C. It is known that bare 2,2’-bithiophene is a crystalline compound with a melting range of 32–33 °C [114]. So, the observed phenomenon could be attributed to the existence of two types of populations of **BTh-OMeOx**, with the crystalline part containing bitiophene most likely differing in size and/or thickness. In the second heating run, the endotherm becomes symmetrical, centered at 44.6 °C, and accompanied by the T_g_ of OMeOx of 69 °C. It turns out that in the cooling cycle of **BTh-OMeOx** (Figure 6), besides the phenomenon of OMeOx vitrification (evidenced by the signal at 61.3 °C), rearrangement of the bithiophenic sequences also took place, giving rise to two consecutive exothermic crystallization peaks centered at 14 °C and −2 °C, respectively.

From these results two main conclusions can be drawn regarding **BTh-OMeOx**. The first one is that, due to its amphiphilic nature, the expected microphase separation took place during its precipitation from the reaction mixture, as confirmed by the presence in the first heating run of the melting endotherm of bitiophene and by the T_g_ of OMeOx. The second conclusion is that microphase separation takes place in the molten-softened state of **BTh-OMeOx** as well. This assertion is supported by the cooling and the second heating runs in Figure 6. A detailed study will be carried out in the future to understand the intimate, reciprocal arrangement of the structural constituents of **BTh-OMeOx** at the molecular level, given that, 2,2’-bithiophene derivatives are very important themselves and as building blocks for synthesis of polymers used in electronics or in the biomedical field.

#### 2.3.2. Self-Acid Assisted Polymerization (SAAP) of **Th-OMeOx** Macromonomer in Bulk

Solid state polymerization (SSP) is an ideal attractive and environmentally sound procedure given that it can be implemented at relatively low operating temperatures in which side reactions and thermal degradation should be insignificant, while requiring inexpensive and uncomplicated equipment [115]. The idea of SSP of a suitable monomer in a well-ordered crystalline state was already realized in the 1960s and 1970s with polydiacetylenes and (SN)x, but SSP of a thiophene derivative was serendipitously discovered and reported by Wudl and Perepichka, who observed that prolonged storage (about two years) of 2,5-dibromo-3,4-ethylenedioxythiophene(DBEDOT) at room temperature gave a bluish conjugated polymer [116]. Over time, other scientists confirmed and employed this C-Br/C-Br polycondensation method [115,117,118,119].

On the other hand, performed in bulk [120,121] or in solution [122,123], the autopolymerization of halogeno-mono-substituted thiophene derivatives, in particular of 2-bromo-substituted thiophenes, was discovered as well [120,121], and a different mechanism than SSP was proposed, [120,121]. Because the generated hydrobromic acid (HBr) or external acid addition would accelerate and catalyze this C-Br/C-H polycondensation process, it was stated that an acid-assisted mechanism may be involved. The first examples of this heat-promoted polycondensation, named self-acid assisted polycondensation (SAAP) [121], were demonstrated in bulk using the oily, brominated 3,4-ethylenedioxythiophene (EDOT) [120,121].

To the best of our knowledge, there are only a few reports about polythiophenes synthesized by SAAP from mono-halogenated monomers using SSP [124,125]. In those studies, the authors investigated in a detailed way the effect of monomer structure on the resulting homopolymer via SAAP. They concluded that, as far as monomer design is concerned, the position of the halogen atoms in relation to the alkyloxy or ethylenedioxy substitutes at the third position of the tiophene ring definitely influence the success of SAAP, and that its close position should be favorable [124]. Taking into account the important role of unsymmetrical molecules in synthesis of multifunctional polythiophenes, in the context of SAAP, which offers the advantages of being solvent- and trace-metal-free, to explore polymerization of **Th-OMeOx** that fulfills the needed structural criteria, was considered an opportunity. 

The procedure for polymerization is described in the Experimental Procedures. 

In order to properly adjust SSP reaction parameters, thermogravimetrical behavior and thermal stability of **Th-OMeOx** was taken into account. It shows good thermal stability (Figure S6A), having a one-step degradation process, with the IDT around 300 °C and the main weight loss (78%), being recorded in the temperature range of 300–450 °C. In our tests we followed not only the influence of the incubation temperature and the incubation time on the obtained products, but also determined if closing the vial affected the reaction pathway (Table 2).

As confirmed by simple naked eye visual observation and supported by FT-IR spectra investigations (data not shown) under the conditions presented in Table 2 and denoted as samples **1** to **5**, for which the used incubation temperatures were equal or only slightly higher than T_g_, after 24 h of incubation, **Th-OMeOx** did not polymerize. Thus, it was clear from these results that a temperature under 150 °C is most probably too low to ensure adequate mobility of OMeOx chains to release the reactive positions of the possibly buried thiophene rings in the tight arrangement of the solid state.

**Figure 7:**
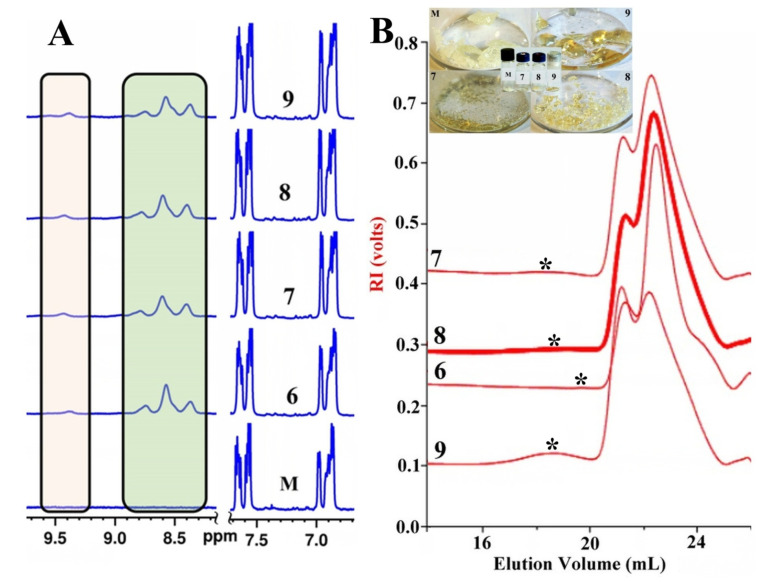
(**A**) ^1^H-NMR spectra in DMSO-d_6_ (the aromatic region) of macromonomer **Th-MeOx** (**M**) and of the resulting mixtures after SAAP in 2, 4 and 6 h of incubation at 150 °C (open and closed vials); (**B**) GPC traces in Chl of the mixtures resulting after SAAP of macromonomer **Th-MeOx**: **M**-macromonomer; **7**: 4 h in open vial; **8**: 4 h in closed vial; **9**: 6 h in open vial; in the inset are photos of the samples in solid state after SAAP.

After several other attempts, it was experimentally found that transformation (eventually polymerization) started to develop at 150 °C, and incubations were performed for different time intervals (Table 2). Glassy, brownish materials were obtained, which changed their aspect in an incubation-time-dependent manner, as can be seen in the photos in Figure 7B. Using FT-IR spectroscopy, by comparatively analyzing the spectrum of **Th-OMeOx** and that of the product obtained after 2 h of incubation, we got the net evidence that thiophene oligomerization took place due to the presence of three small but well-resolved peaks at 1521 cm^−1^, 1542 cm^−1^ and 1559 cm^−1^ (Figure S7). These peaks have a position similar to some of those of **BTh-OMeOx** placed in the spectrum in the same range (vide supra). Therefore, it may be reasonable to conclude that at least dimers formed after 2 h of incubation. This conclusion is also supported by ^1^H-NMR (Figure 7A). The results given by this technique have been combined with GPC measurements (Figure 7B), which are very sensitive to changes in the molecular weight of polymers.

As can be seen in the ^1^H-NMR spectrum of sample **6**, by comparing with the starting **Th-OMeOx**, a new group of signals in the range 8.3–8.8 ppm appeared (greenish area), which were attributed to the protons of the thiophene rings regio-randomly enchained into a conjugated sequence [38]. Moreover, a new, small peak that is discernible in all the treated samples appeared at approximately 9.4 ppm. It could be due to the proton in the hydroxyl of ω-chain-ends of OMeOx, which are pendant as side chains in increasing numbers on the conjugated poly/oligomeric thiophene main chain. This newly appearing group of signals is present for all time intervals of incubation. Besides, in the range 6.9–7.2 ppm, the noticed changes, even if discrete, are in perfect accordance with the configurational complexity (four triads of monomeric units) of the newly formed oligomers, with a chain equal or higher than trimers [38]. The presence in the spectra of the signals before 7 ppm, attributable to the protons belonging to a thiophene ring at the chain’s end (**a** in Figure 1), supports, on the one hand, the conclusion that the reaction was not complete. On the other hand, the existence of short oligomers could also be supported. Because the proton signals of some of the enchained thiophene rings overlap with some of the macromonomer signals [38], it was not possible to accurately calculate the degree of its transformation. A rough estimation was attempted by comparing the intensities of the peaks corresponding to the protons at the fourth position of all the thiophene rings (macromonomer, dimers and longer chains) in the range 6.8–7 ppm (**b** in Figure 1) with the intensity of the signal in the range 8.3–8.8 ppm. The obtained percentages decrease in the order: 21.5% (**6**) >17.2% (**8**) >17% (**7**) >15.5% (**9**). These values induced the conclusion that by increasing the incubation time, the macromonomer’s polymerization chance decreased. 

The conclusion that the reaction was not complete in any of the tested conditions (samples **6** to **9** in Table 2) and instead a mixture of the **Th-OMeOx** macromonomer with newly formed homo/oligopolymers could result from the reaction can be also drawn from the GPC elugrams presented in Figure 7B. From all traces, only sample **6** shows a shoulder at an elution volume higher than 24 mL. However, the general tendency is evident in that as the incubation time increases, the molecular weight of the formed homopolymer also increases, with the maxima of the elugrams shifting from higher elution volumes to smaller ones. In addition, with increasing the reaction time, an increasingly evident new peak at a lower elution volume (marked with asterisk in Figure 7B) is visible; this is evidence of the formation of longer polythiophene chains having an increasing IPD value. Analysis of these peaks gave the following order of M_n_: 20,200 (IPD = 1.36)(**9**) > 19,200 (IPD = 1.29)(**7**) > 18,800 (IPD = 1.36)(**8**). Attempts to increase incubation time did not result in significant improvement in molecular weights, suggesting a threshold at the used temperature.

The experiment of keeping the reaction vial closed was performed as it has been claimed that autopolymerization of 2-bromo-substituted thiophene derivatives is catalytically accelerated by the generated HBr gas [120]. Our results suggest that keeping the formed vaporized HBr inside the vial has a detrimental effect on Mn and IPD. However, if the results of the rough estimation for macromonomer transformation degree are taken into consideration, it was slightly improved by closing the vial. In spite of the caution which generally should be considered, the GPC results for g-CPs, the data from Figure 7B show that the **Th-OMeOx** macromonomer has the ability to polymerize by SAAP at a temperature significantly higher than the T_g_ of the macromonomer, a mixture of oligo/polymers of different length being formed. This is in sharp contrast with SSP of DBEDOT, for which heating above the melting point of the monomer significantly inhibits the reaction [120]. 

However, the viscous state in which the polymerization of **Th-OMeOx** takes place could be one of the possible explanations for the incompletion of the reaction. High viscosity of the medium can limit the mobility of the reactants. Further, favorable intermolecular interactionsmay be slowed down by the “melt-like” state in which the reaction is developing. Consequently, performing polymerization in bulk at a higher temperature than 150 °C in solution (in the presence of Lewis [122] or Bronsted [123] acids) or alternatively, based on its aqueous self-dispersibility, applying direct arylation in an aqueous medium [126] is expected to have better performance creating a polythiophene-g-OMeOx, and these ideas will be explored in future studies. 

## 3. Materials and Methods

### 3.1. Materials

2-Bromo-3-(bromomethyl)thiophene, thiophene 2-boronic acid (Merck-Sigma-Aldrich, Darmstadt, Germany)), 2-methyl-2-oxazoline (MeOX), poly(2-methyl-2-oxazoline), hydroxy terminated,(PMeOx, c.a.) M_n_ = 5000; IPD = 1.3), potassium hydroxide (KOH), sodium bicarbonate (NaHCO_3_) and tetrakis(triphenylphosphine)palladium(0) (Pd(PPh_3_)_4_)(all from Sigma-Aldrich) were used as received. All the solvents were purified and dried by usual methods.

#### 3.1.1. Synthesis of Thiophene-Ended Oligo(2-methyl-2-oxazoline) Macromonomer (**Th-OMeOx**)

**Th-OMeOx** was obtained by cationic ring-opening polymerization (CROP) of 2-methyl-2-oxazoline (MeOX) using 2-bromo-3-(bromomethyl)thiophene (I_1_) as an initiator. Briefly, a solution of MeOX (40.4 mmol, 3.41 ml) and I_1_ (2.7 mmol, 0.35 mL) in dry acetonitrile (24 mL) was kept at 80 °C for 24 h in nitrogen. To end the reaction, about 10 mL of KOH methanolic solution (0.2 M) was added. The as-obtained mixture was stirred at the same temperature for another hour and then cooled down to room temperature. The resulted macromonomer was precipitated in diethyl ether. **Th-OMeOx** purification was achieved by passing its solution in acetone through a silica-gel-filled column and subsequent precipitation in cold diethyl ether to obtain 3.567g (yield = 92%).

#### 3.1.2. Synthesis of 2,2’-3-OMeOx-Substituted Bithiophene Macromonomer (**BTh-OMeOx**) by Suzuki Condensation

Into a 100 mL three neck round bottom flask, equipped with a condenser, a rubber septum, nitrogen inlet-outlet and magnetic stirrer, 20 mL of 1N NaHCO_3_ aqueous solution and 30 mL of DMF were combined, degassed by bubbling nitrogen over a period of 1 h, then refluxed under nitrogen for 3 h.

A 50 mL three neck round bottom flask equipped similarly as the previous one was charged under inert atmosphere with 1.05 mmol (1.57 g) macromonomer **Th-OMeOx**, 2.63 mmol (0.336g) thiophene 2-boronic acid and 0.015 mmol (0.020g) Pd(PPh_3_)_4_. Then 17 mL of 1N NaHCO_3_/DMF homogeneous system was introduced with a syringe through the septum. The reaction was performed for 5 days with vigorous stirring at reflux, under nitrogen atmosphere and with the exclusion of light. After that period, the reaction was stopped, and the water was distilled off by a rotary evaporator. After cooling to room temperature, in order to remove the catalyst and the unreacted reagents, the organic layer was extracted several times with acetonitrile, filtered, concentrated by rotary evaporator and precipitated in cold diethyl. After filtration and drying under vacuum, a white solid was obtained. The product was further passed through a silicagel column using acetonitrile as eluent and reprecipitated in cold diethyl ether. The total yield was 82%.

#### 3.1.3. SAAP of **Th-OMeOx** in Bulk

SAAP of **Th-OMeOx** macromonomer was done similar to the solid-state polymerization (SSP) previously reported [116,124]. Briefly, **Th-OMeOx** (0.02 g) was weighed in glass vials and incubated from 65 °C to 150 °C for different periods (see Table 1). After drying in a vacuum oven at room temperature, glassy-like materials with different degrees of brownish color were formed (see photos in Figure 7) for the **PTh-OMeOx**-containing samples (Scheme 1). 

### 3.2. Measurements

^1^H-NMR spectra were recorded at room temperature on a Bruker Avance NEO −400 spectrometer (400 MHz) in solutions of CDCl_3_, DMSO-d_6_ or D_2_O, and chemical shifts are reported in ppm and referenced to TMS as the internal standard. 

The relative molecular weight and index of polydispersity (IPD) were determined by gel permeation chromatography (GPC) in two different solvents. The measurements in Chl were performed by using a WGE SEC-3010 multidetection system, consisting of a pump, two PL gel columns (PL gel 5 micro Mixed C Agilent and PL gel 5 micro Mixed D Agilent), dual detector refractometer/viscometer (RI/VI) WGE SEC-3010 and flow rate of 1.0 mL/min at 30 °C. The RI/VI detector was calibrated with PS standards (580–467,000 DA) having narrow molecular weight distribution. The system was also equipped with a UV detector WGE SEC-3010 and Bi-MwA Brookhaven multi-angle SLS detector. Data were analyzed using PARSEC Chromatography software. Determination in N,N’-dimethylformamide DMF was performed using a Waters 515 instrument at a flow rate of 0.3 mL min^−1^ and monodisperse polystyrene standards for the calibration plot.

The FTIR spectra were recorded on a Bruker Vertex 70 FTIR spectrometer equipped with a diamond ATR device (Golden Gate, Bruker) in transmission mode by using KBr pellets. 

UV–vis absorption spectra were measured using a Specord 200 Analytik Jena spectrophotometer. Fluorescence measurements were carried out using a Perkin Elmer LS 55 apparatus. All measurements were done in water, Chl, THF and ACN by keeping the concentration of solutions constant at 1 mg/mL.

DSC experiments were conducted on a Perkin Elmer 4000 DSC apparatus calibrated with indium. Around 10 mg of each sample was weighed in pressed and punched aluminum crucibles. Nitrogen was used as inert atmosphere at a flow rate of 50 mL/min. Heating and cooling rates of 40 °C/min and 10 °C/min were applied, respectively. The glass transition temperature (T_g_) was taken as the mid-point on the curve showing the heat capacity change. TGA measurements were performed on Perkin Elmer Diamond TGA/DTA equipment. Around 10 mg of each sample was weighed in alumina crucibles with no lids. A heating rate of 10 °C/min was applied. Nitrogen was used as inert atmosphere at a flow rate of 50 mL/min. Melting (T_m_) and crystallization (T_c_) temperatures were defined as the temperatures corresponding to the maxima of the respective enthalpy peaks, while T_g_ was defined as the half-height of the heat capacity step associated with the glass transition.

Particle characterization was carried out by dynamic light scattering (DLS) using a Malvern Zetasizer Nano ZS instrument equipped with a 4.0 mW He–Ne laser operating at 633 nm and a detection angle of 173°. The intensity weighted mean hydrodynamic size (Z average) and the polydispersity factor were obtained from analysis using the autocorrelation function. Samples were used as prepared, without filtration, at a concentration of c = 1 mg/mL in different solvents. The reported values represent the average of three measurements for each sample at 25 °C with an equilibration time of 5 min before starting each measurement. Quartz cells were used for organic solvents.

The morphology of **Th-OMeOx** in thin film was obtained by atomic force microscopy (AFM) using an NTEGRA Spectra (NT-MDT, Russia) instrument with commercially available silicon nitride cantilevers (NSG10, NT-MDT, Russia) operated in tapping mode under ambient conditions. Silicon cantilever tips (NSG 10) with a resonance frequency of 140–390 kHz, a force constant of 5.5–22.5 Nm^−1^ and 10 nm tip curvature radius were used.

The samples were prepared by drop-casting on freshly cleaved muscovite mica of the dispersions of macromonomer in the given solvents at a concentration of 1 mg/mL. Dried at ambient temperature, in order to allow for uniform evaporation, the samples were kept in a solvent-saturated atmosphere by using a glass dome that only partially covered them. The resulting topographical AFM images were analyzed using Nova 1.0.26.1443 software.

## 4. Conclusions

Using controlled CROP, a new functional and versatile OMeOx-based macromonomer was synthesized, which was designed with a multi-level amphiphilic character (solvophilic, shape, electronic and flexibility anisotropy) by placing at its α-chain-end a 2-Br-Th moiety and at the ω-chain-end a hydroxyl group. 

In addition to basic structural characterization, its capability to work as a reactive building block for other materials with added complexity and functionality was demonstrated. 

Thus, adding an extra thiophene ring to **Th-OMeOx** through Suzuki condensation resulted in **BTh-OMeOx**, which is a unique combination of a rigid and crystallizable π-conjugated sequence with a flexible, oligomeric and amorphous one. **BTh-OMeOx** is also water self-dispersible, shows photoluminescence and, by its oxidative condensation polymerization, is expected to conduct to a more regioregular polythiophene with regularly attached OMeOx side chains due to its unsymmetrical substitution at the third position. Not the least, similar to its precursor **Th-OMeOx**, its self-assembling capability in aqueous media and its fluorescence could be exploited as a potential fluorescent reporter and/or as a fluorescent carrier for various kinds of therapeutics. The availability of the hydroxyl ω-chain-end of OMeOx offers the possibility for post-polymerization functionalization with various biologically significant targeting groups (such as folic acid) for multifunctional theranostics.

Having a 2-bromosubstitute thiophene reactive moiety, **Th-OMeOx**’s SAAP in bulk was attempted, and a mixture of polymer/oligomers and unreacted **Th-OMeOx** was obtained only when the process was conducted at a much higher temperature than its T_g_. The polymerization showed time-sensitiveness and influence due to the retained HBr inside the reaction vial. 

The importance and novelty of our findings rely on the fact that, to the best of our knowledge, this is the first example of SAAP in bulk of a 2-substituted thiophene compound that is not crystalline, in this way enlarging the thiophene-based SSP database. Moreover, based on and with these results in hand, more successful polymerization of **Th-OMeOx** is expected at higher temperature or by applying “in solution” techniques.

The study of **Th-OMeOx** in different solvents highlights the importance of its peculiar amphiphilic structural construction, and more importantly, the impact the introduction of the thiophene ring has on the behavior and properties of OMeOx. Further, it was demonstrated that **Th-OMeOx** can work as a multifunctional material, with the capability to self-assemble in aqueous or organic media, forming nanostructured particles (micelles or vesicles) that behave like clusteroluminogens, showing blue luminescence under a wide range of excitation wavelengths. In thin film obtained from THF dispersion, surprisingly, tubular vesicles of tens of nanometers in length are formed. To the best of our knowledge, this is the first OMeOx-based non-conventional luminophore prepared by conventional CROP for PMeOx synthesis reported to date. By finding the blue luminescence property of **Th-OMeOx**, it was demonstrated that OMeOx is not only a polypeptide conformational isomer but also a functional biomimetic of it, endowing **Th-OMeOx** with a valuable characteristic. All in all, the presented results confirm once again the strength of end-group functionalization for synthesis of polymer-based materials with complex behavior.

## Figures and Tables

**Table 1:** Size of self-assembled structures formed by **Th-OMeOx** in different solvents at various concentrations and their fluorescence data ^c^.

Solvent (conc., mg/mL)	Size of the Self-Assembled Structures (nm) ^a^	λ_max_^em^ (nm) ^b^ (λ_ex_ = 315 nm)	λ_max_^em^ (nm) ^b^ (λ_ex_ = 330 nm)
**water (2)**	**496**	*360* (*sh*); **405**	381; **413**
**water (1.5)**	**278**	*356* (*sh*); **407**	378; **417**
**water (1)**	**251**	**358**; **408**	378; **418**
**water (0.7)**	**248**	**354**; **410**	373; **422**
**water (0.5)**	**520**	**357**; **407**	375; **417**
**Chl (1)**	**627**	**396**; *490* (*sh*); *540* (*sh*)	**400**; **420**; *450* (*sh*); *476* (*sh*)
**THF (1)**	**1.05 (20%)** **875 (70%)** **4428 (10%)**	*349* (*sh*); **365**; *373* (*sh*)	**370**; *382* (*sh*)

^a^—determined by DLS measurements; ^b^—obtained by fluorescence spectroscopy; **^c^**—the concentrations were varied in water; italics indicate emissions as shoulders (*sh*) while bold gives main emission peaks (**λ_max_^em^**).

**Table 2:** The reaction conditions used for SAAP *.

Sample No.	1	2	3	4	5	6	7	8	9
Temperature (°C)	65	80	90	100	130	150	150	150	150
Reaction time (h)	24	24	24	24	4	2	4	4	6

* All experiments were performed in open vials except for no. 8, which was performed in a closed vial.

## Data Availability

The data presented in this study are available on request from the corresponding authors.

## Supplementary Materials

The following supporting information can be downloaded at: https://www.mdpi.com/article/10.3390/ijms23147495/s1. More detail can refer to Refs. [38,46,91,127,128,129,130,131,132,133,134,135,136,137].

## References

[1] Walsha D.J., Guironnet D. (2019). Macromolecules with programmable shape, size, and chemistry. Proc. Natl. Acad. Sci. USA.

[2] Polymeropoulos G., Zapsas G., Ntetsikas K., Bilalis P., Gnanou Y., Hadjichristidis N. (2017). 50th Anniversary Perspective: Polymers with Complex Architectures. Macromolecules.

[3] Hadjichristidis N., Pispas S., Pitsikalis M. (1999). End-functionalized polymers with zwitterionic end-groups. Prog. Polym. Sci..

[4] Lunn D.J., Discekici E.H., Read de Alaniz J., Gutekunst W.R., Hawker C.J. (2017). Established and Emerging Strategies for Polymer Chain-End Modification. J. Polym. Sci. Part A Polym. Chem..

[5] Kim J., Jung H.Y., Park M.J. (2020). End-Group Chemistry and Junction Chemistry in Polymer Science: Past, Present, and Future. Macromolecules.

[6] Zhou D., Zhu L.-W., Wu B.-H., Xu Z.-K., Wan L.-S. (2022). End-functionalized polymers by controlled/living radical polymerizations: Synthesis and applications. Polym. Chem..

[7] Lai Y., Lei Y., Xu X., Li Y., He B., Gu Z. (2013). Polymeric micelles with π-π conjugated cinnamic acid as lipophilic moieties for doxorubicin delivery. J. Mater. Chem. B.

[8] Jiang Y., Hadjichristidis N. (2019). Tetraphenylethene-Functionalized Polyethylene-Based Polymers with Aggregation-Induced Emission. Macromolecules.

[9] Ito K. (1998). Polymeric Design by Macromonomer Technique. Prog. Polym. Sci..

[10] Hadjichristidis N., Pitsikalis M., Iatrou H., Pispas S. (2003). The Strength of the Macromonomer Strategy for Complex Macromolecular Architecture: Molecular, Characterization, Properties and Applications of Polymacromonomers. Macromol. Rapid Commun..

[11] Rempp P., Franta E., Masson P., Lutz P. (1986). Macromonomers as polymeric intermediates. Synthesis and Applications. Progr. Colloid Polym. Sci..

[12] Yamashita Y. (1981). Synthesis and characterization of functional graft copolymers by macromonomer technique. J. Appl. Polym. Sci.Appl. Polym. Symp..

[13] Neugebauer D. (2016). Macromonomers. Encyclopedia of Polymer Science and Technology.

[14] Cianga I., Yagci Y. (2004). New polyphenylene-based macromolecular architectures by using well defined macromonomers synthesized via controlled polymerization methods. Prog. Polym. Sci..

[15] Müllner M., Müller A.H.E. (2016). Cylindrical polymer brushes—Anisotropic building blocks, unimolecular templates and particulate nanocarriers. Polymer.

[16] Xie G., Martinez M.R., Olszewski M., Sheiko S.S., Matyjaszewski K. (2019). Molecular Bottlebrushes as Novel Materials. Biomacromolecules.

[17] Zhang X., Dai Y. (2019). Recent development of brush polymers via polymerization of poly(ethylene glycol)-based macromonomers. Polym. Chem..

[18] Pizzi D., Humphries J., Morrow J.P., Fletcher N.L., Bell C.A., Thurecht K.J., Kempe K. (2019). Poly(2-oxazoline) macromonomers as building blocks for functional and biocompatible polymer architectures. Eur. Polym. J..

[19] Alkan S., Toppare L., Hepuzer Y., Yagci Y. (1999). Block Copolymers of Thiophene-Capped Poly(methyl methacrylate) with Pyrrole. J. Polym. Sci. Part A Polym. Chem..

[20] Mecerreyes D., Pomposo J.A., Bengoetxea M., Grande H. (2000). Novel Pyrrole End-Functional Macromonomers Prepared by Ring-Opening and Atom-Transfer Radical Polymerizations. Macromolecules.

[21] Simionescu C.I., Grovu-Ivanoiu M., Cianga I., Grigoras M., Duca A., Cocarla I. (1996). Electrochemical polymerization of some monomers with Schiff’s base structure. Angew. Makromol. Chem..

[22] Cianga I., Yagci Y. (2001). Polystyrene macromonomer with boronic acid propanediol diester functionality prepared by ATRP for synthesis of comb-like polyphenylenes. Polym. Bull..

[23] Cianga I., Yagci Y. (2002). Synthesis and characterization of comb-like polyphenylenes via Suzuki coupling of polystyrene macromonomers prepared by atom transfer radical polymerization. Eur. Polym. J..

[24] Papila O., Toppare L., Yagci Y., Cianga L. (2004). Conducting Copolymers of Thiophene-Functionalized Polystyrene. Int. J. Polym. Anal. Charact..

[25] Sahin E., Camurlu P., Toppare L., Mercore V.M., Cianga I., Yagci Y. (2005). Synthesis and characterization of thiophene functionalized polystyrene copolymers and their electrochemical properties. Polym. Int..

[26] Colak D.G., Cianga I., Yagci Y., Cirpan A., Karasz F.E. (2007). Novel poly(phenylene vinylenes) with well-defined poly(ε-caprolactone) or polystyrene as lateral substituents: Synthesis and characterization. Macromolecules.

[27] Wang Y., Park J.S., Leech J.P., Miao S., Bunz U.H.F. (2007). Poly(aryleneethynylenes) with Orange, Yellow, Green, and Blue Solid-State Fluorescence. Macromolecules.

[28] Molina B.G., Bendrea A.D., Cianga L., Armelin E., del Valle L.J., Cianga I., Alemán C. (2017). The biocompatible polythiophene-g-polycaprolactone copolymer as an efficient dopamine sensor platform. Polym. Chem..

[29] Molina B.G., Bendrea A.-D., Lanzalaco S., Franco L., Cianga L., del Valle L.J., Puiggali J., Turon P., Armelin E., Cianga I. (2020). Smart design for a flexible, functionalized an electroresponsive hybrid platform based on poly(3,4-ethylenedioxythiophene) derivatives to improve cell viability. J. Mater. Chem. B.

[30] Marina S., Mantione D., Manoj Kumar K., Kari V., Gutierrez J., Tercjak A., Sanchez-Sanchez A., Mecerreyes D. (2018). New electroactive macromonomers and multi-responsive PEDOT graft copolymers. Polym. Chem..

[31] Dominguez-Alfaro A., Gabirondo E., Alegret N., De León-Almazán C.M., Hernandez R., Vallejo-Illarramendi A., Prato M., Mecerreyes D. (2021). 3D Printable Conducting and Biocompatible PEDOT-graft-PLA Copolymers by Direct Ink Writing. Macromol. Rapid Commun..

[32] Waugaman M., Sannigrahi B., McGeady P., Khan I.M. (2003). Synthesis, characterization and biocompatibility studies of oligosiloxane modified polythiophenes. Eur. Polym. J..

[33] Ohnishi I., Hashimoto K., Tajima K. (2018). Synthesis of diketopyrrolopyrrole-based polymers with polydimethylsiloxane side chains and their application in organic field-effect transistors. R. Soc. Open Sci..

[34] Fruth A., Klapper M., Mullen K. (2010). Synthesis and Characterization of Amphiphilic Polyelectrolyte Brush Copolymers Based on Poly(2,7-carbazole). Macromolecules.

[35] Demir B., Yilmaz T., Guler E., Pinar Gumus Z., Akbulut H., Aldemir E., Coskunol H., Colak D.G., Cianga I., Yamada S. (2016). Polypeptide with electroactive endgroups as sensing platform for the abused drug ‘methamphetamine’ by bioelectrochemical method. Talanta.

[36] Shi H., Wang Y., Huang X., Liang P., Tang Y., Zhang Y., Fu N., Huang W., Dong X. (2018). NIR-Absorbing water-soluble conjugated polymer dots for photoacoustic imaging-guided photothermal/photodynamic synergetic cancer therapy. J. Mater. Chem. B.

[37] Molina B.G., Cianga L., Bendrea A.-D., Cianga I., del Valle L.J., Estrany F., Aleman C., Armelin E. (2018). Amphiphilic polypyrrole-poly(Schiff base) copolymers with poly(ethylene glycol) side chains: Synthesis, properties and applications. Polym. Chem..

[38] Cianga L., Bendrea A.-D., Fifere N., Nita L.E., Doroftei F., Ag D., Seleci M., Timur S., Cianga I. (2014). Fluorescent micellar nanoparticles by self assembly of amphiphilic, nonionic and water self-dispersible polythiophenes with “hairy rod” architecture. RSC Adv..

[39] Bendrea A.-D., Cianga L., Hitruc E.G., Titorencu I., Cianga I. (2013). Fluorescent Nanoparticles from “Hairy-Rods”, Water-Self Dispersible Amphiphilic Polythiophenes. Mater. Plast..

[40] Bendrea A.-D., Fabregat G., Cianga L., Estrany F., del Valle L.J., Cianga I., Aleman C. (2013). Hybrid materials consisting of an all-conjugated polythiophene backbone and grafted hydrophilic poly(ethylene glycol) chains. Polym. Chem..

[41] Yuksel M., Colak D.G., Akin M., Cianga I., Kukut M., Medine E.I., Can M., Sakarya S., Unak P., Timur S. (2012). Nonionic, Water Self-Dispersible “Hairy-Rod” Poly(p-phenylene)-g-poly(ethylene glycol) Copolymer/Carbon Nanotube Conjugates for Targeted Cell Imaging. Biomacromolecules.

[42] Bendrea A.-D., Cianga L., Ailiesei G.-L., Ursu E.-L., Colak D.G., Cianga I. (2021). 3,4-Ethylenedioxythiophene (EDOT) End-Group Functionalized Poly-ε-caprolactone (PCL): Self-Assembly in Organic Solvents and Its Coincidentally Observed Peculiar Behavior in Thin Film and Protonated Media. Polymers.

[43] Yang Y., Liu Z., Zhang G., Zhang X., Zhang D. (2019). The Effects of Side Chains on the Charge Mobilities and Functionalities of Semiconducting Conjugated Polymers beyond Solubilities. Adv. Mater..

[44] Huang H., Liao Y., Bu W., Wang W., Sun J.Z. (2016). Going beyond the classical amphiphilicity paradigm: The self-assembly of completely hydrophobic polymers into free-standing sheets and hollow nanostructures in solvents of variable quality. Soft Matter.

[45] Colak D.G., Cianga I., Cianga L., Yagci Y. (2016). Synthesis and self-assembly of fluorenevinylene alternating copolymers in “Hairy-Rod” architecture: Side chain-mediated tuning of conformation, microstructure and photophysical properties. Des. Monomers Polym..

[46] Molina B.G., Cianga L., Bendrea A.-D., Cianga I., Alemán C., Armelin E. (2019). An amphiphilic, heterografted polythiophene copolymer containing biocompatible/biodegradable side chains for use as an (electro)active surface in biomedical applications. Polym. Chem..

[47] Colak D.G., Egbe D.A.M., Birckner E., Yurteri S., Cianga I., Tekin E., Schubert U.S., Yagci Y. (2009). Photophysical properties of PPP and PPV derivatives bearing polystyrene or polycaprolactone as side groups. Eur. Polym. J..

[48] Uyar T., Cianga I., Cianga L., Besenbacher F., Yagci Y. (2009). Self-aligned and bundled electrospun fibers prepared from blends of polystyrene (PS) and poly(methyl methacrylate) (PMMA) with a hairy-rod polyphenylene copolymer. Mater. Lett..

[49] Kallitsis J.K., Andreopoulou A.K., Moller M., Matyjaszewski K. (2012). Rigid-Flexible and Rod-Coil Copolymers, Macromolecular Architectures and Soft Nano-Objects. Polymer Science: A Comprehensive Reference.

[50] Xu Z., Park K.S., Diao Y. (2020). What Is the Assembly Pathway of a Conjugated Polymer From Solution to Thin Films?. Front. Chem..

[51] Hicks G.E.J., Li S., Obhi N.K., Jarrett-Wilkins C.N., Seferos D.S. (2021). Programmable Assembly of π-Conjugated Polymers. Adv. Mater..

[52] Mullin W.J., Sharber S.A., Thomas S.W. (2021). Optimizing the self-assembly of conjugated polymers and small molecules through structurally programmed non-covalent control. J. Polym. Sci..

[53] Sahin E., Camurlu P., Toppare L., Mercore V.M., Cianga I., Yagci Y. (2005). Conducting copolymers of thiophene functionalized polystyrenes with thiophene. J. Electroanal. Chem..

[54] Kurosawa T., Chiu Y.-C., Zhou Y., Gu X., Chen W.-C., Bao Z. (2016). Impact of Polystyrene Oligomer Side Chains on Naphthalene Diimide–Bithiophene Polymers as n-Type Semiconductors for Organic Field-Effect Transistors. Adv. Funct. Mater..

[55] Wen H.-F., Wu H.-C., Aimi J., Hung C.-C., Chiang Y.-C., Kuo C.-C., Chen W.-C. (2017). Soft Poly(butyl acrylate) Side Chains toward Intrinsically Stretchable Polymeric Semiconductors for Field-Effect Transistor Applications. Macromolecules.

[56] Du W., Ohayon D., Combe C., Mottier L., Maria I.P., Ashraf R.S., Fiumelli H., Inal S., McCulloch I. (2018). Improving the Compatibility of Diketopyrrolopyrrole Semiconducting Polymers for Biological Interfacing by Lysine Attachment. Chem. Mater..

[57] Baek P., Aydemir N., An Y., Chan E.W.C., Sokolova A., Nelson A., Mata J.P., McGillivray D., Barker D., Travas-Sejdic J. (2017). Molecularly Engineered Intrinsically Healable and Stretchable Conducting Polymers. Chem. Mater..

[58] Luo S.-C. (2013). Conducting Polymers as Biointerfaces and Biomaterials: A Perspective for a Special Issue of Polymer Reviews. Polym. Rev..

[59] Baek P., Voorhaar L., Barker D., Travas-Sejdic J. (2018). Molecular Approach to Conjugated Polymers with Biomimetic Properties. Acc. Chem. Res..

[60] Bendrea A.-D., Cianga L., Cianga I. (2011). Review paper: Progress in the Field of Conducting Polymers for Tissue Engineering Applications. J. Biomater. Appl..

[61] Petty A.J., Keate R.L., Jiang B., Ameer G.A., Rivnay J. (2020). Conducting Polymers for Tissue Regeneration in vivo. Chem. Mater..

[62] Wei J., Liu Y., Yu J., Chen L., Luo M., Yang L., Li P., Li S., Zhang X.-H. (2021). Conjugated Polymers: Optical Toolbox for Bioimagingand Cancer Therapy. Small.

[63] Borges-Gonzalez J., Kousseff C.J., Nielsen C.B. (2019). Organic semiconductors for biological sensing. J. Mater. Chem. C.

[64] Sista P., Ghosh K., Martinez J.S., Rocha R.C. (2014). Polythiophenes in Biological Applications. J. Nanosci. Nanotechnol..

[65] So R.C., Carreon-Asok A.C. (2019). Molecular Design, Synthetic Strategies, and Applications of Cationic Polythiophenes. Chem. Rev..

[66] Zangoli M., DiMaria F. (2021). Synthesis, characterization, and biological applications of semiconducting polythiophene-based nanoparticles. View.

[67] Kim H.-C., Kim E., Lee S.G., Lee S.J., Jeong S.W., Ha T.-L., Lee B., Lee S.W. (2016). Folic Acid-Functionalized Polythiophene for Targeted Cellular Imaging. J. Nanosci. Nanotechnol..

[68] Guler E., Akbulut H., Geyik C., Yilmaz T., Gumus Z.P., Barlas F.B., Ahan R.E., Demirkol D.O., Yamada S., Endo T. (2016). Complex Structured Fluorescent Polythiophene Graft Copolymer as a Versatile Tool for Imaging, Targeted Delivery of Paclitaxel, and Radiotherapy. Biomacromolecules.

[69] Bendrea A.-D., Fabregat G., Torras J., Maione S., Cianga L., del Valle L.J., Cianga I., Aleman C. (2013). Polythiophene-g-poly(ethylene glycol) graft copolymers for electroactive scaffolds. J. Mater. Chem. B.

[70] Maione S., Fabregat G., del Valle L.J., Bendrea A.-D., Cianga L., Cianga I., Estrany F., Aleman C. (2015). Effect of the Graft Ratio on the Properties of Polythiophene-g-poly(ethylene glycol). J. Polym. Sci. Part B Polym. Phys..

[71] Perez-Madrigal M.M., Cianga L., del Valle L.J., Cianga I., Aleman C. (2015). Electroactive and bioactive films of random copolymers containing terthiophene, carboxyl and Schiff base functionalities in the main chain. Polym. Chem..

[72] Aydin M., Aydin E.B., Sezginturk M.K. (2019). A Highly Selective Poly(thiophene)-graft-Poly(methacrylamide) Polymer Modified ITO Electrode for Neuron Specific Enolase Detection in Human Serum. Macromol. Biosci..

[73] Lorson T., Lübtow M.M., Wegener E., Haider M.S., Borova S., Nahm D., Jordan R., Sokolski-Papkov M., Kabanov A.V., Luxenhofer R. (2018). Poly(2-oxazoline)s based biomaterials: A comprehensive and critical update. Biomaterials.

[74] Vlassi E., Papagiannopoulos A., Pispas S. (2017). Amphiphilic poly(2-oxazoline) copolymers as self-assembled carriers for drug delivery applications. Eur. Polym. J..

[75] Akbar M., Cagli E., Erel-Göktepe I. (2019). Layer-By-Layer Modified Superparamagnetic Iron Oxide Nanoparticles with Stimuli Responsive Drug Release Properties. Macromol. Chem. Phys..

[76] You Y., Kobayashi K., Colak B., Luo P., Cozens E., Fields L., Suzuki K., Gautrot J. (2021). Engineered cell-degradable poly(2-alkyl-2-oxazoline) hydrogel for epicardial placement of mesenchymal stem cells for myocardial repair. Biomaterials.

[77] Cagli E., Ugur E., Ulusan S., Banerjee S., Erel-Goktepe I. (2019). Effect of side chain variation on surface and biological properties of poly(2-alkyl-2-oxazoline) multilayers. Eur. Polym. J..

[78] Grube M., Leiske M.N., Schubert U.S., Nischang I. (2018). POx as an Alternative to PEG? A Hydrodynamic and Light Scattering Study. Macromolecules.

[79] Lubich C., Allacher P., de la Rosa M., Bauer A., Prenninger T., Horling F.M., Siekmann J., Oldenburg J., Scheiflinger F., Reipert B.M. (2016). The Mystery of Antibodies Against Polyethylene Glycol (PEG)—What do we Know?. Pharm. Res..

[80] Benski L., Tiller J.C. (2019). Telechelic biocidal poly(2-oxazoline)s and polycations. Eur. Polym. J..

[81] Cirpan A., Alkan S., Toppare L., David G., Yagci Y. (2001). Synthesis and electroactivity of pyrrole-functionalized poly(2-methy-2-oxazoline). Eur. Polym. J..

[82] Wang Y., Wilson J.N., Smith M.D., Bunz U.H.F. (2004). TEMPO-Substituted PPEs: Polystyrene-PPE Graft Copolymers and Double Graft Copolymers. Macromolecules.

[83] Demirel L.A., Yurteri S., Cianga I., Yagci Y. (2005). Layered Morphology of Poly(phenylene)s in Thin Films Induced by Substitution of Well-Defined Poly(ε-caprolactone) Side Chains. Macromolecules.

[84] Cianga I., Mercore V.M., Grigoras M., Yagci Y. (2007). Poly(thienyl-phenylene)s with macromolecular side chains by oxidative polymerization of well-defined macromonomers. J. Polym. Sci. Part A Polym. Chem..

[85] Demirel L.A., Yurteri S., Cianga I., Yagci Y. (2007). Synthesis and Morphological Characterization of Poly(ϵ-caprolactone) and Poly(2-methyl-oxazoline) substituted Phenyl Rings and Phenylene Oligomers. J. Polym. Sci. Part A Polym. Chem..

[86] Wang Y., Andrew J.Z., Wilson J.N., Kim I.-B., Solntsev K.M., Tolbert L.M., Bunz U.H.F. (2008). Optical Spectroscopy of Grafted Poly(p-phenyleneethynylene)s in Water and Water-DMF Mixtures. Macromolecules.

[87] Alemseghed M.G., Servello J., Hundt N., Sista P., Biewer M.C., Stefan M.C. (2010). Amphiphilic Block Copolymers Containing Regioregular Poly(3-hexylthiophene) and Poly(2-ethyl-2-oxazoline. Macromol. Chem. Phys..

[88] Chan E.W.C., Baek P., De la Rosa V.R., Barker D., Hoogenboom R., Travas-Sejdic J. (2017). Thermoresponsive laterally-branched polythiophene phenylene derivative as water-soluble temperature sensor. Polym. Chem..

[89] Creamer A., Wood C.S., Howes P.D., Casey A., Cong S., Marsh A.V., Godin R., Panidi J., Anthopoulos T.D., Burgess C.H. (2018). Post-polymerisationfunctionalisation of conjugated polymer backbones and its application in multifunctional emissive nanoparticles. Nat. Commun..

[90] Lam K.H., Foong T.R.B., Ooi Z.E., Zhang J., Grimsdale A.C., Lam Y.M. (2013). Enhancing the Performance of Solution-Processed Bulk-Heterojunction Solar Cells Using Hydrogen-Bonding-Induced Self-Organization of Small Molecules. ACS Appl. Mater. Interfaces.

[91] Jin R.-H., Motoyoshi K.-I. (1999). Porphyrin-centered Water-soluble Star-shaped Polymers: Poly(*N*-acetylethylenimine) and Poly(ethylenimine) Arms. J. Porphyr. Phthalocyanines.

[92] Bose A., Jana S., Saha A., Mandal T.K. (2017). Amphiphilic polypeptide-polyoxazoline graft copolymer conjugate with tunable thermoresponsiveness: Synthesis and self-assembly into various micellar structures in aqueous and nonaqueous media. Polymer.

[93] Heller L.E., Whitleigh J., Roth D.F., Oherlein E.M., Lucci F.R., Kolonko K.J., Plass K.E. (2012). Self-Assembly of Isomeric Monofunctionalized Thiophenes. Langmuir.

[94] Glassner M., Vergaelen M., Hoogenboom R. (2018). Poly(2-oxazoline)s: A comprehensive overview of polymer structures and their physical properties. Polym. Int..

[95] Yuan W.Z., Zhang Y. (2017). Nonconventional Macromolecular Luminogens with Aggregation-Induced Emission Characteristics. J.Polym. Sci. Part A Polym. Chem..

[96] Tomalia D.A., Klajnert-Maculewicz B., Johnson K.A.-M., Brinkman H.F., Janaszewska A., Hedstrand D.M. (2019). Non-traditional intrinsic luminescence: Inexplicable blue fluorescence observed for dendrimers, macromolecules and small molecular structures lacking traditional/conventional luminophores. Prog. Polym. Sci..

[97] Zhang Z., Zhang Z., Zhang H., Sun J.Z., Tang B.Z. (2022). The mysterious blue emission around 440 nm in carbonyl-based aliphatic clusteroluminogens. J. Polym. Sci..

[98] Wang Z., Zhang H., Li S., Lei D., Tang B.Z., Ye R. (2021). Recent Advances in Clusteroluminescence. Top. Curr. Chem..

[99] Liao P., Huang J., Yan Y., Tang B.Z. (2021). Clusterization-triggered emission (CTE): One for all, all for one. Mater. Chem. Front..

[100] Jiang N., Zhu D., Su Z., Bryce M.R. (2021). Recent advances in oligomers/polymers with unconventional chromophores. Mater. Chem. Front..

[101] Tang S., Yang T., Zhao Z., Zhu T., Zhang Q., Hou W., Yuan W.Z. (2021). Nonconventional luminophores: Characteristics, advancements and perspectives. Chem. Soc. Rev..

[102] Zhang H., Tang B.Z. (2021). Through-Space Interactions in Clusteroluminescence. JACS Au.

[103] Zhou X., Luo W., Nie H., Xu L., Hu R., Zhao Z., Qin A., Tang B.Z. (2017). Oligo(maleic anhydride)s: A platform for unveiling the mechanism of clusteroluminescence of non-aromatic polymers. J. Mater. Chem. C.

[104] Zhou Q., Wang Z., Dou X., Wang Y., Liu S., Zhang Y., Yuan W.Z. (2019). Emission mechanism understanding and tunable persistent room temperature phosphorescence of amorphous nonaromatic polymers. Mater. Chem. Front..

[105] Naumann C.A., Brooks C.F., Fuller G.G., Lehmann T., Ruhe J., Knoll W., Kuhn P., Nuyken O., Frank C.W. (2001). Two-Dimensional Physical Networks of Lipopolymers at the Air/Water Interface: Correlation of Molecular Structure and Surface Rheological Behavior. Langmuir.

[106] Kroning A., Furchner A., Adam S., Uhlmann P., Hinrichs K. (2016). Probing carbonyl-water hydrogen-bond interactions in thin polyoxazoline brushes. Biointerphases.

[107] del Mercato L.L., Pompa P.P., Maruccio G., Torre A.D., Sabella S., Tamburro A.M., Cingolani R., Rinaldi R. (2007). Charge transport and intrinsic fluorescence in amyloid-like fibrils. Proc. Natl. Acad. Sci. USA.

[108] Bonifácio V.D.B., Correia V.G., Pinho M.G., Lima J.C., Aguiar-Ricardo A. (2012). Blue emission of carbamic acid oligooxazoline biotags. Mat. Lett..

[109] Du J., Willcock H., Patterson J.P., Portman I., O’Reilly R.K. (2011). Self-Assembly of Hydrophilic Homopolymers: A Matter of RAFT End Groups. Small.

[110] Rettler E.F.-J., Lambermont-Thijs H.M.L., Kranenburg J.M., Hoogenboom R., Unger M.V., Siesler H.W., Schubert U.S. (2011). Water uptake of poly(2-N-alkyl-2-oxazoline)s: Influence of crystallinity and hydrogen-bonding on the mechanical properties. J. Mater. Chem..

[111] Kolodziejczyk B., Mayevsky D., Winther-Jensen B. (2013). Enhanced absorption spectra of conducting polymers co-polymerised from thiophene derivatives. RSC Adv..

[112] Hoffmann K.J., Bakken E., Samuelsen E.J., Carlsen P.H.J. (2000). Synthesis and polymerization of 3,3′-dialkyl-2,2′-bithiophenes. Synth. Met..

[113] Ogoshi T., Kim K.-M., Chujo Y. (2003). Synthesis and characterization of transparent poly(2-methyl-2-oxazoline) (POZO)-vanadium oxide (V_2_O_5_) hybrids with reversible formation. J. Mater. Chem..

[114] https://www.sigmaaldrich.com/RO/en/product/aldrich/241636.

[115] Pisuchpena T., Keaw-on N., Kitikulvarakorn K., Kusonsong S., Sritana-ananta Y., Supaphol P., Hovena V.P. (2017). Electrospinning and solid state polymerization: A simple and versatile route to conducting PEDOT composite films. Eur. Polym. J..

[116] Meng H., Perepichka D.F., Wudl F. (2003). Facile Solid-State Synthesis of Highly Conducting Poly(ethylenedioxythiophene). Angew. Chem. Int. Ed..

[117] Spencer H.J., Berridge R., Crouch D.J., Wright S.P., Giles M., McCulloch I., Coles S.J., Hursthouse M.B., Skabara P.J. (2003). Further evidence for spontaneous solid-state polymerization reactions in 2,5-dibromothiophene derivatives. J. Mater. Chem..

[118] Patra A., Wijsboom Y.H., Leitus G., Bendikov M. (2011). Tuning the Band Gap of Low-Band-Gap Polyselenophenes and Polythiophenes: The Effect of the Heteroatom. Chem. Mater..

[119] Gulprasertrat N., Chapromma J., Aree T., Sritana-anant Y. (2015). Synthesis of functionalizable derivatives of 3,4 ethylenedioxythiophene and their solid-state polymerizations. J. Appl. Polym. Sci..

[120] Wagner P., Jolley K.W., Officer D.L. (2011). Why Do Some Alkoxybromothiophenes Spontaneously Polymerize?. Aust. J. Chem..

[121] Yin Y., Li Z., Jin J., Tusy C., Xia J. (2013). Facile synthesis of poly(3,4-ethylenedioxythiophene) by acid-assisted polycondensation of 5-bromo-2,3-dihydro-thieno[3,4-b][1,4]dioxine. Synth. Met..

[122] Bonillo B., Swager T.M. (2012). Chain-Growth Polymerization of 2-Chlorothiophenes Promoted by Lewis Acids. J. Am. Chem. Soc..

[123] Balasubramanian A., Ku T.-C., Shih H.-P., Suman A., Lin H.-J., Shih T.-W., Han C.-C. (2014). Chain-growth cationic polymerization of 2-halogenated thiophenes promoted by Brønsted acids. Polym. Chem..

[124] Tusy C., Jiang K., Peng K., Huang L., Xia J. (2015). Effect of monomers’ structure on self-acid assisted polycondensation for the synthesis of poly(3,4 ethylenedioxythiophene) and homopolythiophene. Polym. Chem..

[125] Jiang K., Cai X., Liu X., Xia J. (2019). Exploring functionalized polythiophene derivatives based on thiophene-linker-thiophene platform, analysis of prototype monomer crystal for C-Br/C-H bulk polycondensation and its application for acid detection. Polymer.

[126] Lin Y.-J., Sun H.-S., Yang H.-R., Lai Y.-Y., Hou K.-Y., Liu Y.-H. (2020). Aqueous Palladium-Catalyzed Direct Arylation Polymerization of 2 Bromothiophene Derivatives. Macromol. Rapid Commun..

[127] ACCU DYNE TEST. https://www.accudynetest.com/solubility_table.html?sortby=sort_h_h#007.

[128] Hansen C.M. (2007). Hansen Solubility Parameteres: A User’s Handbook.

[129] Lübtow M.M., Haider M.S., Kirsch M., Klisch S., Luxenhofer R. (2019). Like Dissolves Like? A Comprehensive Evaluation of Partial Solubility Parameters to Predict Polymer-Drug Compatibility in Ultrahigh Drug-Loaded Polymer Micelles. Biomacromolecules.

[130] Yang Z., Yuan Y., Jiang R., Fu N., Lu X., Tian C., Hu W., Fan Q., Huang W. (2014). Homogeneous near-infrared emissive polymeric nanoparticles based on amphiphilic diblock copolymers with perylene diimide and PEG pendants: Self-assembly behavior and cellular imaging application. Polym. Chem..

[131] Boccia A.C., Lukes V., Eckstein-Andicsova A., Kozma E. (2020). Solvent- and concentration-induced self-assembly of an amphiphilic perylene dye. New J. Chem..

[132] Walderhaug H., Söderman O. (2009). NMR studies of block copolymer micelles. Curr. Opin. Colloid Interface Sci..

[133] Heald C.R., Stolnik S., Kujawinski K.S., De Matteis C., Garnett M.C., Illum L., Davis S.S., Purkiss S.C., Barlow R.J., Gellert P.R. (2002). Poly(lactic acid)-Poly(ethylene oxide) (PLA-PEG) Nanoparticles: NMR Studies of the Central Solid-like PLA Core and the Liquid PEG Corona. Langmuir.

[134] Park J.M., Kim Y.J., Jang W.D. (2020). Multimodal Stimuli-Responsive Fluorophore-Functionalized Heterotelechelic Poly(2-isopropyl-2-oxazoline). ACS Appl. Polym. Mater..

[135] Rayeroux D., Travelet C., Lapinte V., Borsali R., Robin J.J., Bouilhac C. (2017). Tunable amphiphilic graft copolymers bearing fatty chains and polyoxazoline: Synthesis and self-assembly behavior in solution. Polym. Chem..

[136] Volet G., Auvray L., Amiel C. (2009). Monoalkyl Poly(2-methyl-2-oxazoline) Micelles. A Small-Angle Neutron Scattering Study. J. Phys. Chem. B.

[137] Volet G., Chanthavong V., Wintgens V., Amiel C. (2005). Synthesis of Monoalkyl End-Capped Poly(2-methyl-2-oxazoline) and Its Micelle Formation in Aqueous Solution. Macromolecules.

