# Supplementary material for: Thiophene α-Chain-End-Functionalized Oligo(2-methyl-2-oxazoline) as Precursor Amphiphilic Macromonomer for Grafted Conjugated Oligomers/Polymers and as a Multifunctional Material with Relevant Properties for Biomedical Applications
Source: Int J Mol Sci. 2022 Jul 6;23(14):7495. doi: 10.3390/ijms23147495 (PMC9317439; doi:10.3390/ijms23147495)
Supplement: Supplementary file 1 [file ijms-23-07495-s001.zip › ICianga-Supplementary Materials- Revised 2.pdf]

Supplementary Materials

Thiophene α-Chain-End - Functionalized Oligo(2-methyl-2-oxazoline) as Precursor Amphiphilic Macromonomer for Grafted Conjugated Oligomers / Polymers and as a Multifunctional Material with Relevant Properties for Biomedical Applications

Anca-Dana Bendrea[1], Luminita Cianga[1]*, Gabriela-Liliana Ailiesei[2], Demet Goen Colak[3], Irina Popescu[4] and Ioan Cianga[1]*

[1] Centre of Advanced Research in Bionanoconjugates and Biopolymers, "Petru Poni" Institute of Macromolecular Chemistry, 41 A, Grigore-Ghica Voda Alley, 700487, Iasi, Romania; anca.bendrea@icmpp.ro (A.-D.B)

[2] NMR Spectroscopy Department, "Petru Poni" Institute of Macromolecular Chemistry, 41 A, Grigore-Ghica Voda Alley, 700487, Iasi, Romania; gdarvaru@icmpp.ro (G.-L. A)

[3] Department of Chemistry, Faculty of Science and Letters, Istanbul Technical University, Maslak, 34469, Istanbul, Turkey; goende@itu.edu.tr (D.G.C)

[4] Department of Natural Polymers, Bioactive and Biocompatible Materials, "Petru Poni" Institute of Macromolecular Chemistry, 41 A, Grigore-Ghica Voda Alley, 700487, Iasi, Romania; ipopescu@icmpp.ro (I.P.)

Corespondence to: ioanc@icmpp.ro (I.C.) and lcianga@icmpp.ro (L.C.)

1. Supplementary Scheme and Figures
2. Supplementary Tables
3. Additional discussion related to the NMR structural characterization
4. Additional discussion regarding the properties in water of commercially available PMeOx hydroxy terminated

4. Supplementary References

1. Scheme and Figures

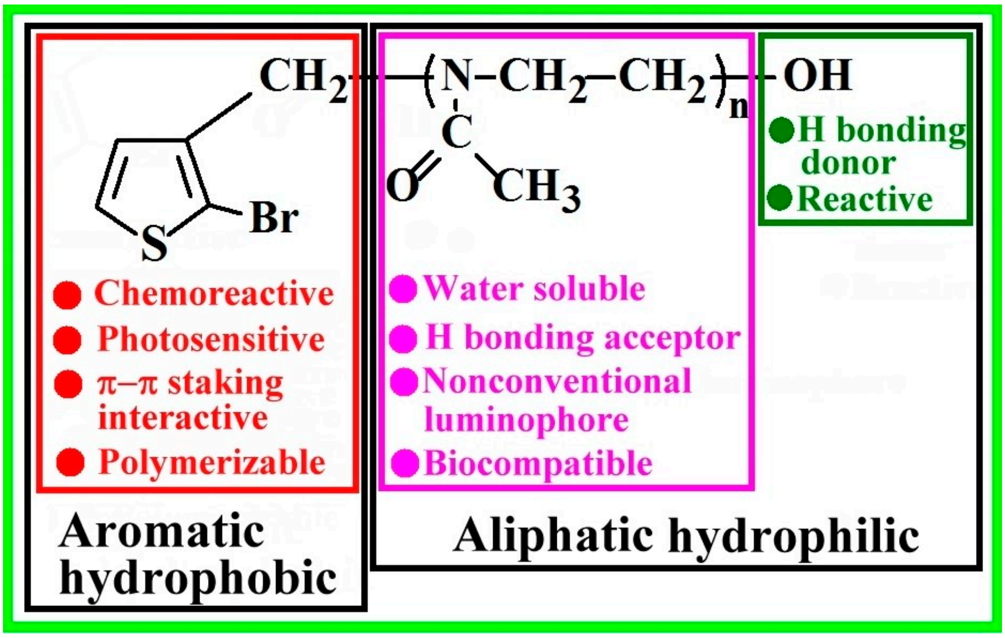

Scheme S1. The design criteria of the amphiphilic macromonomer **Th-OMeOx**

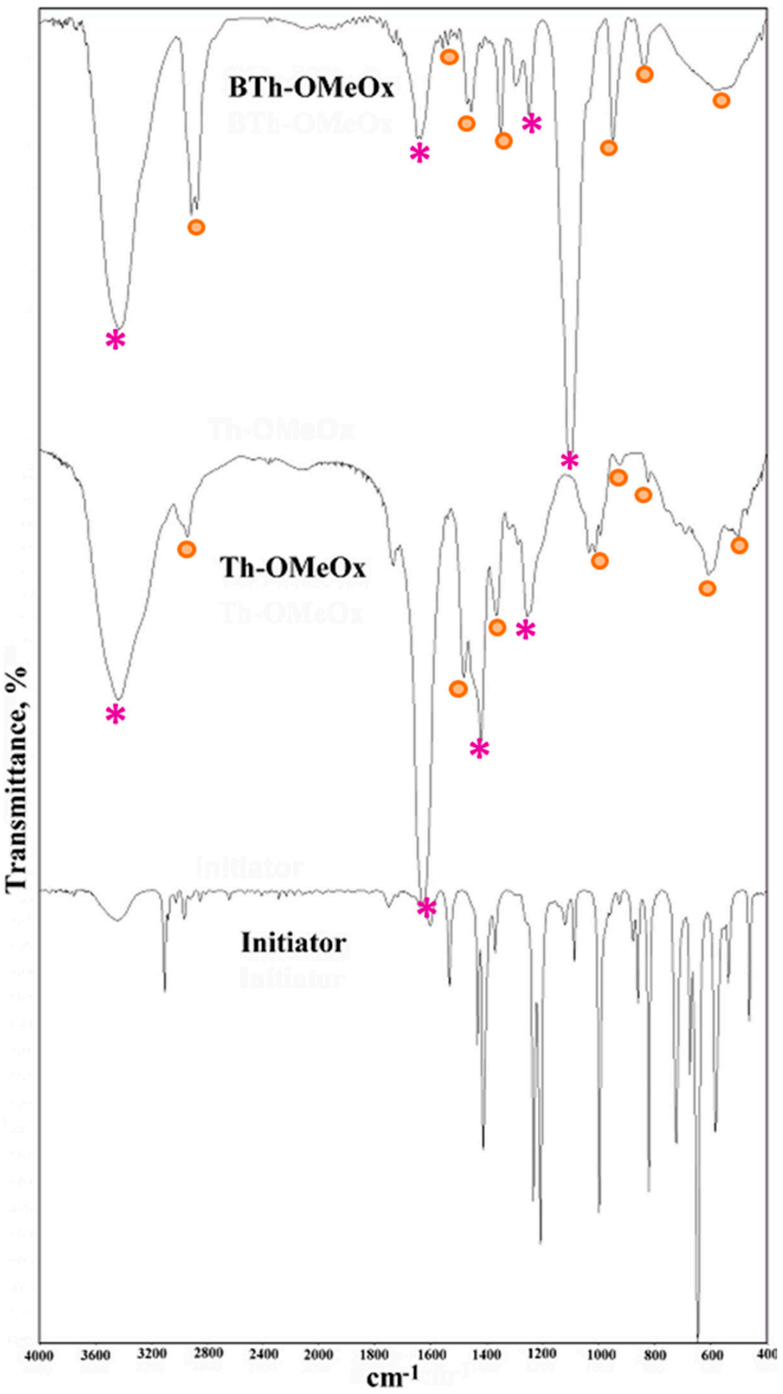

Figure S1. The IR spectra of the initiator (2-bromo-3-(bromomethyl)thiophene), and of the synthesized macromonomers **Th-OMeOx** and **BTh-OMeOx;** * marks the characteristics vibrations of OMeOx, and ● those of aromatic moieties, respectively.

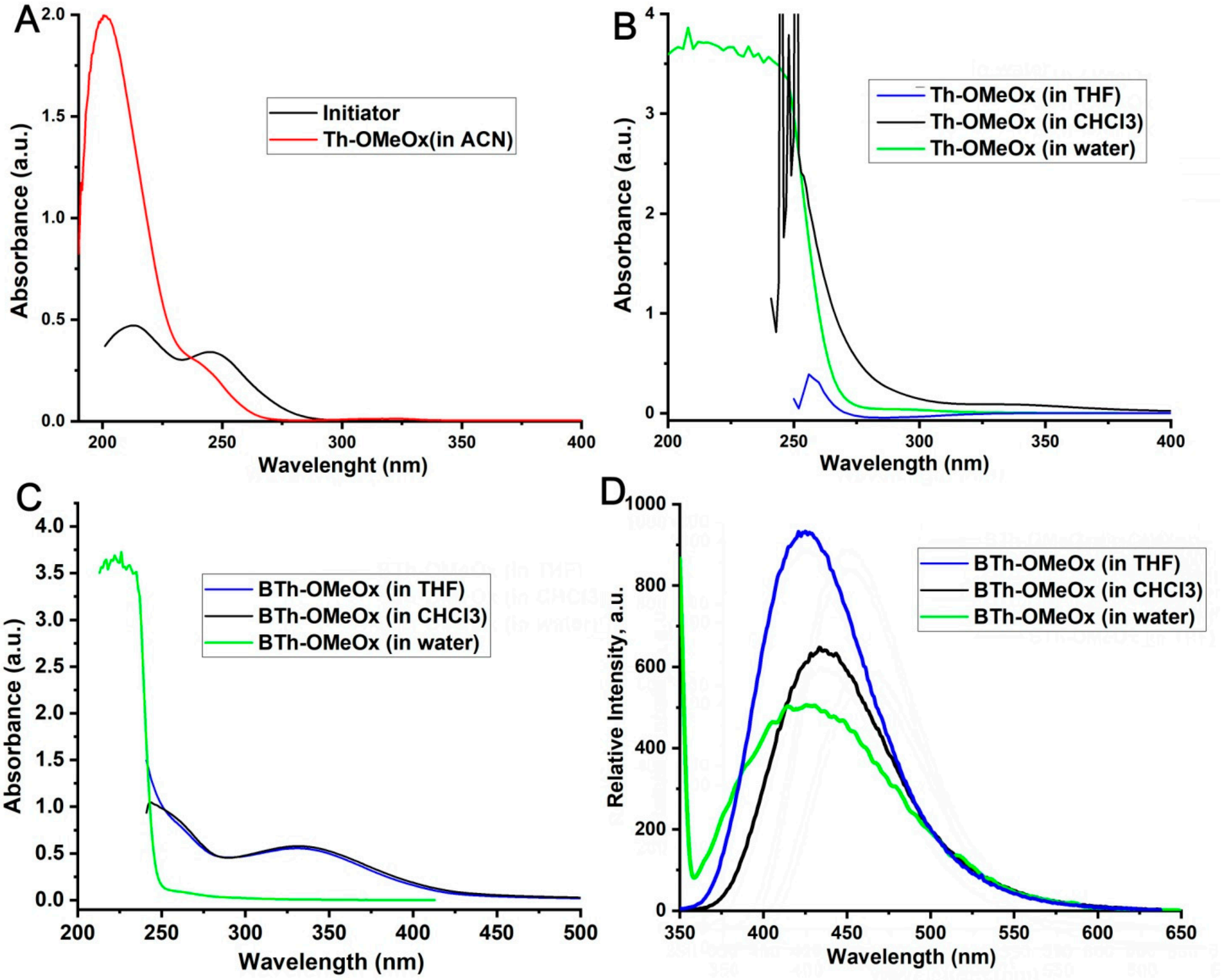

Figure S2. (A)- UV-vis curves of initiator and **Th-OMeOx** in ACN; (B)- UV-vis traces of **Th-OMeOx** in different solvents at c= 1mg/mL; (C)- UV-vis traces of **BTh-OMeOx** in different solvents at c=1 mg/mL; (D)- Florescence measurements of **BTh-OMeOx** at c= 1mg/mL and λ_{ex}= 330nm.

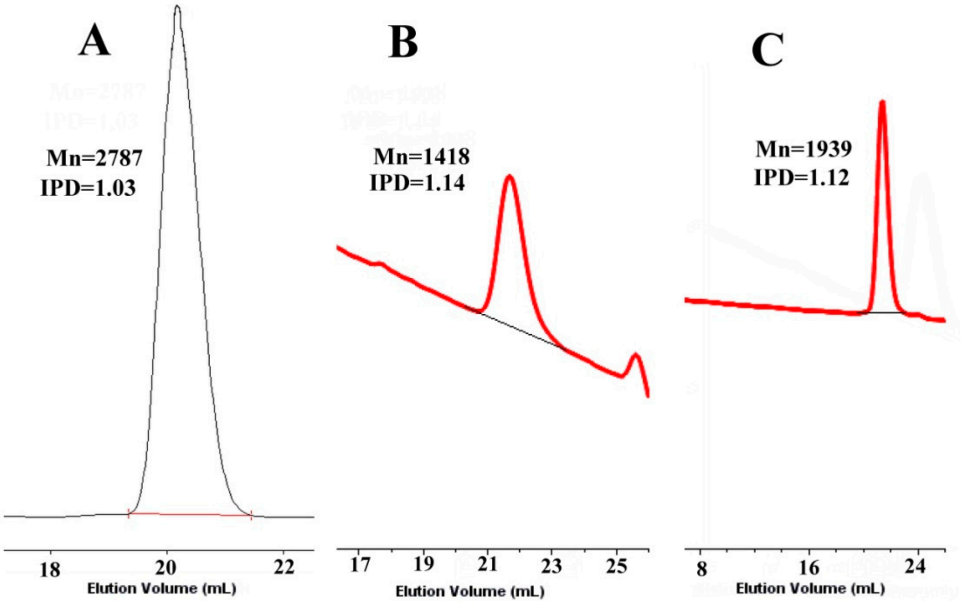

Figure S3. GPC traces of macromonomer **Th-OMeOx** in DMF (**A**) and in Chl (**B**); (**C**)- GPC trace in Chl of macromonomer **BTh-OMeOx**.

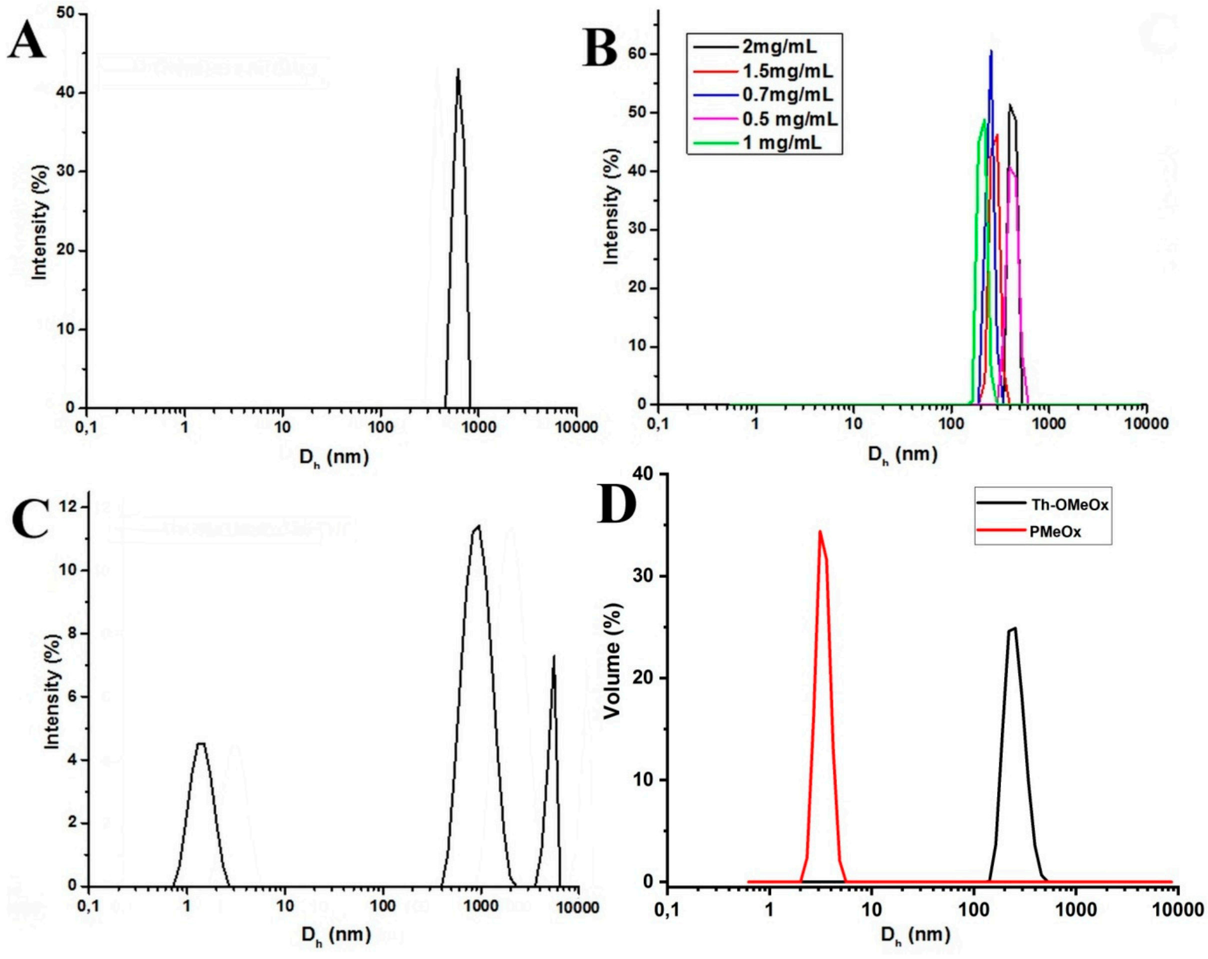

Figure S4. DLS traces of intensity-weighted distribution of apparent hydrodynamic diameter (D_h) for **Th-OMeOx** in Chl at concentration of 1mg/mL (**A**), in water at different concentrations (**B**) and in THF at concentration of 1mg/mL (**C**); Superposition of size distribution of apparent hydrodynamic diameter (D_h) for **Th-OMeOx** and of commercially available **PMeOx** in water at concentration of 1mg/mL (**D**).

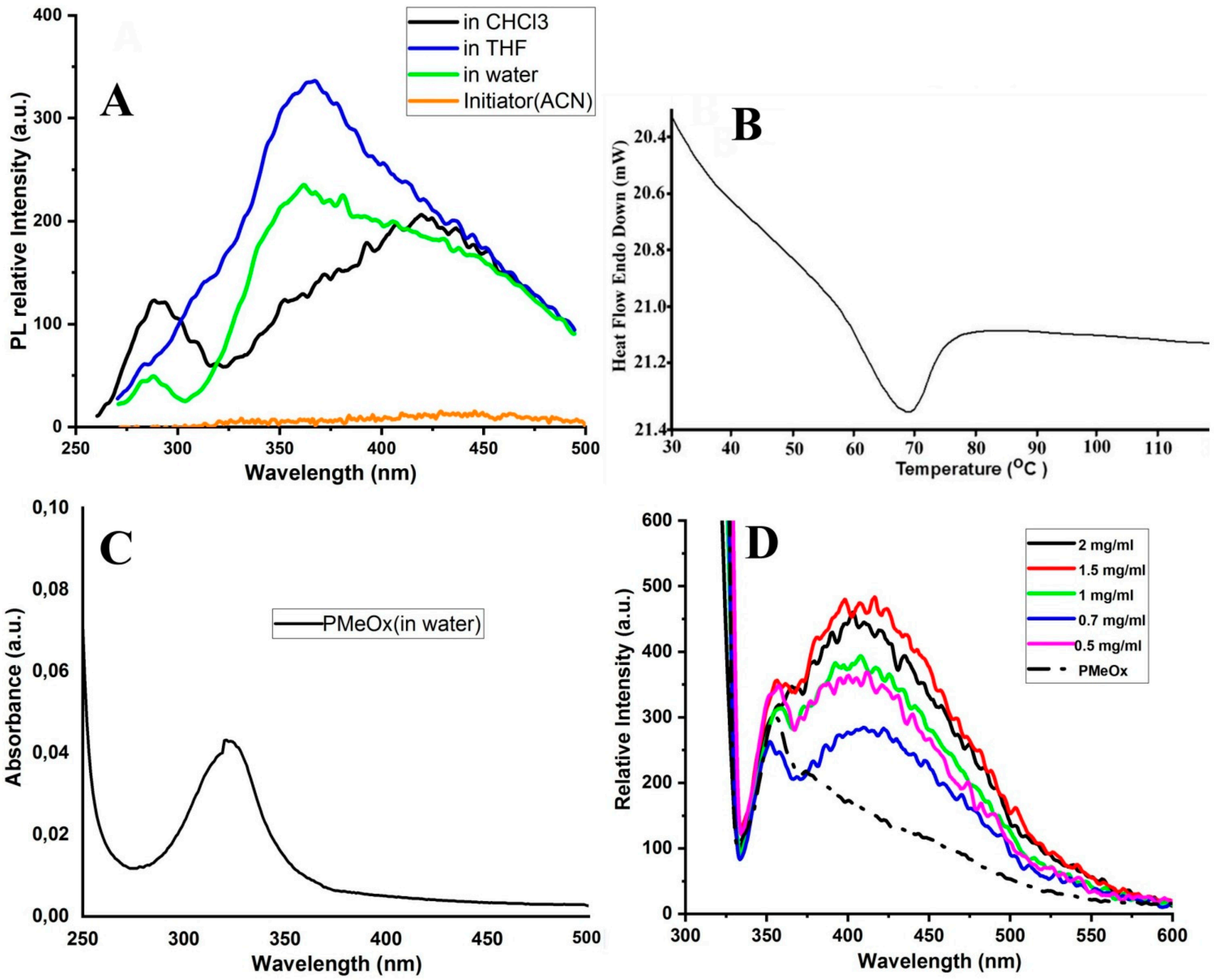

Figure S5. (**A**)- Fluorescence traces of **Th-OMeOx** and of the Th-containing initiator in ACN solution (λ_{ex}= 330nm); (**B**) DSC trace of **Th-OMeOx** in the range 30-140ºC; (C)-UV-vis curve of commercially available **PMeOx** at c= 1mg/mL; (D) Fluorescence curves of **Th-OMeOx** at different concentrations and of commercially available **PMeOx** at c=1 mg/mL in water as solvent registered with λ_{ex}= 315 nm.

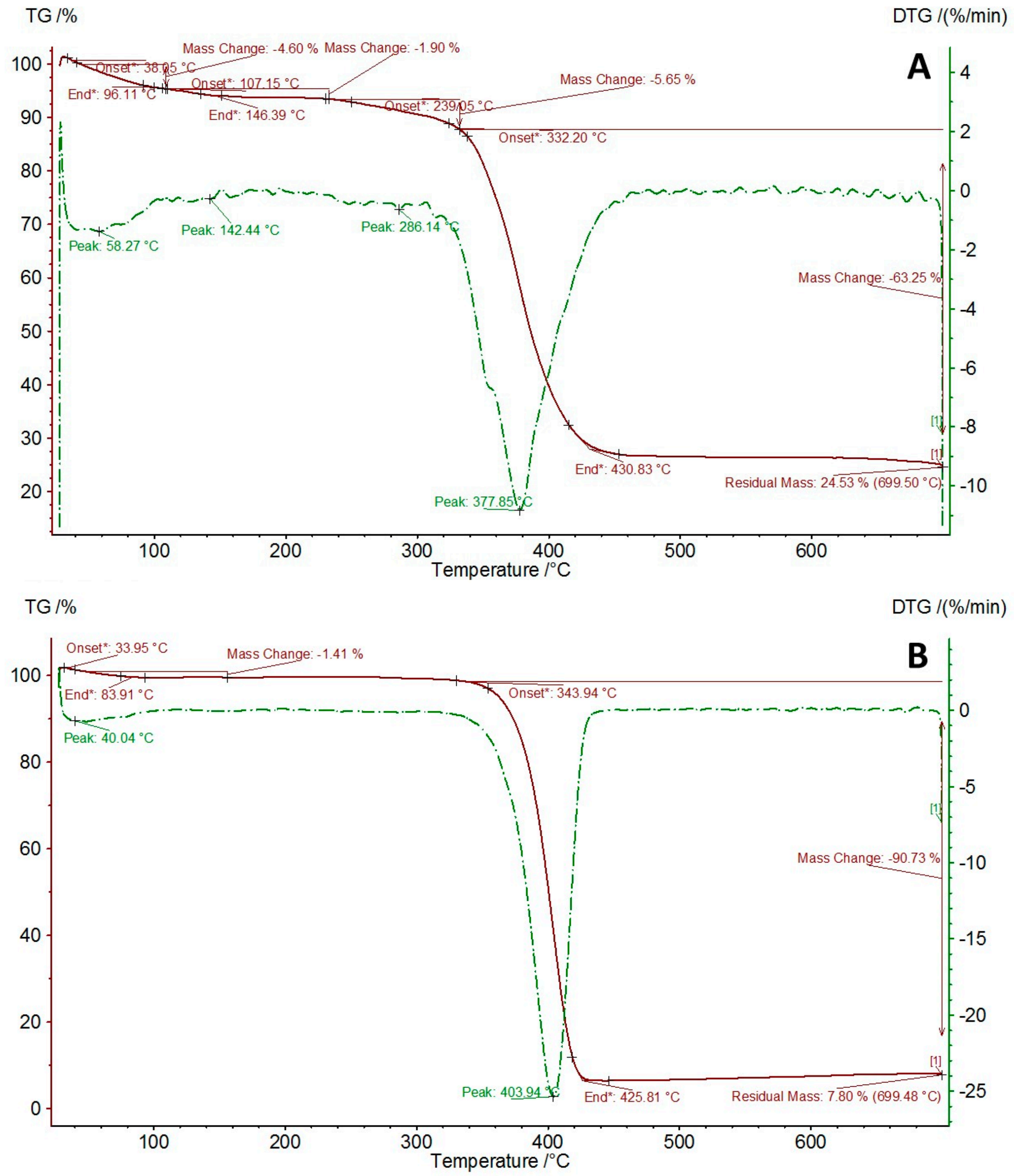

Figure S6. (**A**)- TGA measurement of **Th-OMeOx**; (**B**)- TGA measurement of **BTh-OMeOx** (the registration conditions are given in Experimental Part)

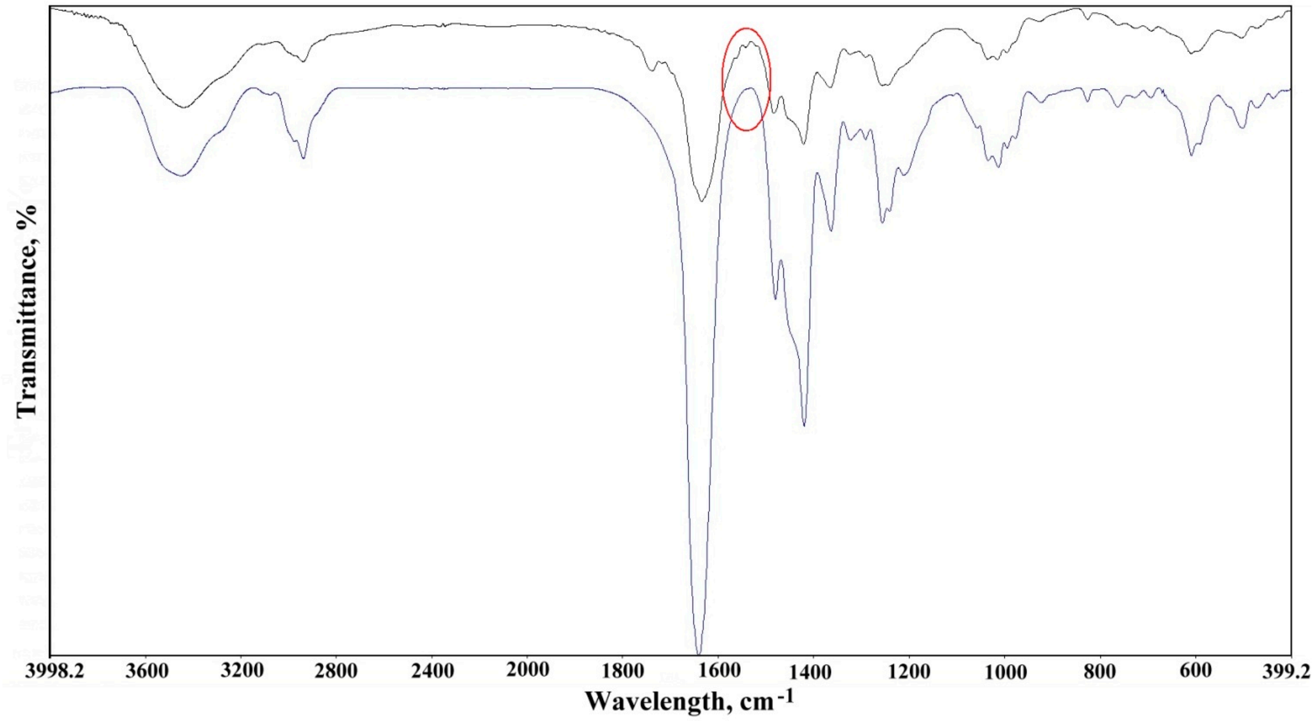

Figure S7. The IR spectra of **Th-OMeOx** (blue line) and of the product resulted after 2 h of its incubation at 150ºC (black line).

2. Tables

Table S1. Some physical properties of the used solvents and those related to the constitutive parts of Th-OMeOx

Compound	Dielectric constant (e)	Hansen Solubility Parameter(MPa)$^{1/2}$				Reference
		δ_t	δ_D	δ_P	δ_H	
**Chl**	4.81	19	17.7	3.1	5.7	1
**THF**	7.52	19.4	16.8	9	8	1
**Water**	80	47.8	15.6	16	42.7	1
**2Br-Th**	-	21.26	20.1	5.2	4.6	2
**PMeOx**	-	24	17	14	10	3

Chl-choloroform; **THF**- tetrahydrofuran; **2Br-Th**- 2-bromothiophene; **PMeOx**-poly(2-methyl-2-oxazoline)

Table S2. The values of λ_{max}^{abs} of Th-OMeOx and of BTh-OMeOx as they appear in the curves in Figure S2 B and C^{a}and of the λ_{max}^{em} from Figure 3A and Figure S2 D

Solvent	Th-OMeOx (λ in nm)		BTh-OMeOx (λ in nm)	
	λ_{max}^{abs}	λ_{max}^{emb}	λ_{max}^{abs}	$\lambda_{max}^{em\ c}$
**Water**	216; 296; 315	288;362; 420(*sh*)	226; 306	425
**THF**	252; 315	284(*sh*);312(*sh*); 366;414(*sh*)	264 (*sh*); 332	425
**Chl**	252; 330	291;420	249; 264 (*sh*); 332	435

a-By UV-vis measurement at a concentration of 1mg/mL; **b**-λ_{ex}=260nm; **c**-λ_{ex}=330nm; *sh*-shoulders of the curves

3. Additional discussion related to the NMR structural characterization

It is well established that the NMR spectroscopy is an important tool for detecting the formation and structures of micelles in solutions of amphiphilic copolymers, of different architecture, in water and in organic solvents, as well [4-8]. When this type of self-assembled structures are formed, because the parts forming the core lose their mobility, the intensity of their signals decreases or disappears completely from the spectrum [4, 7]. While this is an effective mean to probe the selective solvent-induced self-assembling [4-6], however if the chosen solvent for the NMR registration it is not equally good for all the components of the investigated compound, then the micelles formation cannot be ruled out; and using only NMR spectroscopy for structural characterization it is not enough. The micelles formation impacts all the characteristics of the spectrum [9], thus hindering the clear proofing of obtaining the anticipated structure by this technique.

As is the case with the **Th-OMeOx** macromonomer and its **BTh-OMeOx** derivative. They are not only solvophilic amphiphiles but also rigidity and “shape” amphiphiles as well. As such, it is difficult to find a suitable solvent that works as a good solvent for the two disymmetrical parts of these compounds. In this situation, the deviation from the expected theoretically ideal characteristics of the spectra (both ^{1}H-NMR and ^{13}C-NMR) were detected (Figures **1**, **2** and **5**). The impact of the solvent nature effect can be seen in Figure 1, and is more obvious for the hydrophobic aromatic part. Thus, the signals characteristic to the thiophene protons **a** and **b** are significantly different as intensity when comparing with those from OMeOx. Moreover, instead of the expected doublets, two broadened signals can be observed for these protons. An interesting situation is for the methylene -CH2 moiety (which shows proton **c** in Figure 1) and for which instead of the expected singlet, multiple and complicated peaks in shape are detectable in both non-polar $CDCl_3$ and polar DMSO-d_6. This particular behavior could be due to the higher mobility of the -CH_2 when comparing with that of thiophene ring, which allow its probable location, in both solvents, at the core-shell interface of the formed micelles. The multiplet signals splitting feature for proton **c** besides the broad nature suggests that -CH_2 exists in a number of different chemical environments within this relatively interfacial region. This may be due to the different proximities created by the short oxazoline oligomer. A similar impact has been observed in the literature for copolymers of low molecular weight [9].

The short length of OMeOx also allow the appearance of the signals characterizing the terminal structural units separately from the main ones. This particularity give rise to the presence of three peaks (**f**, **f**$_1$ and **f**$_2$) specific for three types of carbonyl function and related three types of methyl groups characterized by **h**, **h**$_1$, and **h**$_2$ carbon atoms in ^{13}C-NMR spectrum in Figure 2. The appearance of three types of signals for the carbon atom in the carbonyl group has recently been reported for a poly(2-isopropyl-2-oxazoline) having aromatic TPE as α- chain end [10]. Another influence factor that could be considered for the deviation from the standard behavior is the polydispersity. It was recognized as factor influencing the copolymers NMR behavior [8]. Even though the polydispersity value of the OMeOx measured by GPC is low, by combining with the short length of the chain, both contributed to the noticed peculiar behavior of **Th-OMeOx** in NMR registration. For this reason, the additional results given by the other performed investigations, in particular of DSC measurements, are welcome in order to complete the NMR results.

The results of DSC in Figure 6, in particular those for **BTh-OMeOx**, for which the only single T_m from bithiophene and T_g from OMeOx are present, confirm that a compound with a unitary structure resulted after Suzuki condensation, with no traces of unreacted thiophene boronic acid. Also the T_g showed by **Th-OMeOx** is practically coincidental with that of **BTh-OMeOx**. This is an indirect evidence that **Th-OMeOx**, as the starting reagent for **BTh-OMeOx** prepartion, has the claimed structure.

4. Additional discussion including the properties in water of commercially available PMeOx hydroxy terminated

To gain a clearer picture of the behavior of the newly synthesized macromonomer **Th-OMeOx** in the aqueous medium (ultrapure Milli-Q water) as a relevant one for biological applications and, at the same time, to evaluate the impact of the insertion of the 2-bromo-substituted thiophene ring (2-Br-Th) as the α end of the OMeOx chain, several investigations were performed using commercially available PMeOx as a model molecule. Thus, it was interesting to follow if PMeOx has the capability for eventually forming self-assembled structures in water (which is known as a good solvent of it) at the same concentration of 1 mg/mL. The result of the DLS measurement, presented in Figure S4 D, confirmed the *a priori* supposition that the polymer, having as α-chain end the methyl group (CH3-), at this

concentration is fully dissolved in water as unimers. Indeed, the experimentally obtained value for hydrodynamic diameter (D_H) of 3.36 nm is corroborated by the reported value of 3.1 nm, calculated for a linear chain of PMeOx containing 32 structural units [11], assuming that PMeOx behaves as a semiflexible chain [12]. Moreover, in a previous report it was shown that the polarity index of pyrene microenvironment remained constant at the value of 1.9 (characteristic for aqueous environment) as long as PMeOx does not form the micellar structures whatever the concentration of it [13].

Therefore, the result for **Th-OMeOx** in Figure S4 D demonstrates an obvious different behavior, essentially given by the presence in its structure of the aromatic thiophene ring which induces an asymmetric amphiphilic character, enabling the micelles' formation due to spontaneous self-assembling. On the other hand, the aqueous solution of PMeOx showed differences of the UV-Vis absorption (Figure S5 C) and of the fluorescence emission (Figure S5 D), when comparing with those of the macromonomer. It can be seen from the Figure S5 C that PMeOx absorption curve shows a first shoulder around 315 nm and a peak at 325 nm due to carbonyl group, a behavior that was previously noticed in the literature [14]. In the fluorescence trace of PMeOx, presented in Figure S5 D, only one emission peak appeared at 357 nm attributable to the emission of the well-solvated pendant acyl groups of the tertiary amide. This is the most relevant evidence that the blue luminescence occurrence for **Th-OMeOx** is due to its micellization in water, resulting in OMeOx clusterization in the confined space of the micelles shell (see the **Figure 3C a** for details). It can be concluded that particular and different behavior of **Th-OMeOx** by comparison with PMeOx is exclusively due to the insertion of the thiophene ring as the OMeOx chain end.

4. References

1. https://www.accudynetest.com/solubility_table.html?sortby=sort_h_h#007 accesed 09 of May 2022.

2. C.M. Hansen, Hansen Solubility Parameteres: A User's Handbook, Secon Ed., CRC Press, 2007, by Taylor & Francis Group, LLC, Boca Raton, Fl 33487-2742, Apendix A: Table A1. pp 361.

3. Michael M. Lubtow, Malik Salman Haider, Marius Kirsch, Stefanie Klisch, and Robert Luxenhofer, Like Dissolves Like? A Comprehensive Evaluation of Partial Solubility

Parameters to Predict Polymer-Drug Compatibility in Ultrahigh Drug-Loaded Polymer Micelles, Biomacromolecules 2019, 20, 3041−3056.

4. Zhen Yang, Yan Yuan, Rongcui Jiang, Nina Fu, Xiaomei Lu, Congcong Tian, Wenbo Hu, Quli Fan, Wei Huang, Homogeneous near-infrared emissive polymeric nanoparticles based on amphiphilic diblock copolymers with perylene diimide and PEG pendants: self-assembly behavior and cellular imaging application, *Polym. Chem*., **2014**, 5, 1372–1380.

5. Boccia, A. C.; Lukes, V.; Eckstein-Andicsova, A.; Kozma, E. Solvent- and concentration-induced self-assembly of an amphiphilic perylene dye. *New J. Chem*. **2020**, 44, 892–899.

6. Cianga, L.; Bendrea, A.-D.; Fifere, N.; Nita, L.E.; Doroftei, F.; Ag, D.; Seleci, M.; Timur, S.; Cianga, I. Fluorescent micellar nanoparticles by self assembly of amphiphilic, nonionic and water self-dispersible polythiophenes with "hairy rod" architecture. *RSC Adv*. **2014**, *4*, 56385–56405.

7. Molina, B. G.; Cianga, L.; Bendrea, A.-D.; Cianga, I.; Alemán, C.; Armelin, E. An amphiphilic, heterografted polythiophene copolymer containing biocompatible/ biodegradable side chains for use as an (electro)active surface in biomedical applications. *Polym. Chem*. **2019**, *10*, 5010–5022.

8. H. Walderhaug , O. Söderman, NMR studies of block copolymer micelles, *Curr. Opin Colloid Interface Sci*. **2009**, 14, 171–177.

9. C. R. Heald, S. Stolnik, K. S. Kujawinski, C. De Matteis, M. C. Garnett, L. Illum,†S. S. Davis, S. C. Purkiss, R. J. Barlow, P. R. Gellert, Poly(lactic acid)-Poly(ethylene oxide) (PLA-PEG) Nanoparticles: NMR Studies of the Central Solid-like PLA Core and the Liquid PEG Corona, *Langmuir* **2002,** *18,* 3669-3675.

10. Jong Min Park, Ye Ji Kim, and Woo-Dong Jang, Multimodal Stimuli-Responsive Fluorophore-Functionalized Heterotelechelic Poly(2-isopropyl-2-oxazoline), ACS Appl. Polym. Mater. 2020, 2, 3535−3542.

11. David Rayeroux, Christophe Travelet, Vincent Lapinte, Redouane Borsali, Jean-Jacques Robin, Cécile Bouilhac, Tunable amphiphilic graft copolymers bearing fatty chains and polyoxazoline: synthesis and self-assembly behavior in solution, Polym. Chem., 2017, *8*, 4246–4263.

12. Gisele Volet, Loic Auvray, and Catherine Amiel, Monoalkyl Poly(2-methyl-2-oxazoline) Micelles. A Small-Angle Neutron Scattering Study, *J. Phys. Chem. B* 2009**,** *113,* 13536–13544.

13. Gisele Volet, Vidalay Chanthavong, Veronique Wintgens, Catherine Amiel, Synthesis of Monoalkyl End-Capped Poly(2-methyl-2-oxazoline) and Its Micelle Formation in Aqueous Solution, *Macromolecules* 2005, *38,* 5190-5197.

14. Jin, R.-H.; Motoyoshi, K.-I. Porphyrin-centered Water-soluble Star-shaped Polymers: Poly(*N*-acetylethylenimine) and Poly(ethylenimine) Arms. *J. Porphyrins Phthalocyanines*. 1999, *3*, 60–64.
